# Neural alignment during face‐to‐face spontaneous deception: Does gender make a difference?

**DOI:** 10.1002/hbm.25173

**Published:** 2020-08-18

**Authors:** Mei Chen, Tingyu Zhang, Ruqian Zhang, Ning Wang, Qing Yin, Yangzhuo Li, Jieqiong Liu, Tao Liu, Xianchun Li

**Affiliations:** ^1^ School of Psychology and Cognitive Science Shanghai Changning‐ECNU Mental Health Center, East China Normal University Shanghai China; ^2^ School of Management Zhejiang University Hangzhou China

**Keywords:** deception, eye contact, gender difference, interpersonal neural synchronization

## Abstract

This study investigated the gender differences in deception and their neural basis in the perspective of two‐person neuroscience. Both male and female dyads were asked to perform a face‐to‐face spontaneous sender–receiver deception task, while their neural activities in the prefrontal cortex (PFC) and right temporal parietal junction (rTPJ) were recorded simultaneously using functional near‐infrared spectroscopy (fNIRS)‐based hyperscanning. Male and female dyads displayed similar deception rate, successful deception rate, and eye contact in deception trials. Moreover, eye contact in deception trials was positively correlated with the success rate of deception in both genders. The fNIRS data showed that the interpersonal neural synchronization (INS) in PFC was significantly enhanced only in female dyads when performed the deception task, while INS in rTPJ was increased only in male dyads. Such INS was correlated with the success rate of deception in both dyads. Granger causality analysis showed that no significant directionality between time series of PFC (or rTPJ) in each dyad, which could indicate that sender and receiver played equally important role during deception task. Finally, enhanced INS in PFC in female dyads mediated the contribution of eye contact to the success rate of deception. All findings in this study suggest that differential patterns of INS are recruited when male and female dyads perform the face‐to‐face deception task. To our knowledge, this is the first interbrain evidence for gender difference of successful deception, which could make us a deeper understanding of spontaneous face‐to‐face deception.

## Graphical abstract

For female dyads, the increased interpersonal neural synchronization (INS) was found in prefrontal cortex during deception. For male dyads, the increased INS was found in right temporal parietal junction during deception.

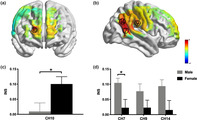

## 1 INTRODUCTION

Deception is a social behavior that one person deliberately tries to mislead others, in order to gain benefits or avoid losses (Abe, 2009; DePaulo et al., 2003). Lies occur frequently on many occasions, such as family, school, workplace, and people tell lies once or twice a day (DePaulo, Kashy, Kirkendol, Wyer, & Epstein, 1996; Edelman & Larkin, 2015). Because of importance to individual development and social stability, deception has drawn rather considerable interest in recent years (Alempaki, Doğan, & Saccardo, 2019; Garrett, Lazzaro, Ariely, & Sharot, 2016; Suchotzki, Verschuere, van Bockstaele, Ben‐Shakhar, & Crombez, 2017; Van Swol & Paik, 2017).

Previous findings have revealed the gender difference in deception in terms of motivation (DePaulo et al., 1996; Meyers‐Levy & Loken, 2015), tactics (Tooke & Camire, 1991; Vasconcellos et al., 2019) and frequency (Capraro, 2017). Males usually display more self‐centered lies to increase their own benefits (DePaulo et al., 1996; Friesen & Gangadharan, 2012). They usually lie to win the favor of females through false promises and spurious ability to obtain resources. While interacting with the same gender, they always tend to exaggerate their advantages, sexual ability, and popularity (Vasconcellos et al., 2019). However, females tell more other‐oriented lies to protect others' interests or comfort others to foster intimacy (DePaulo et al., 1996; Vasconcellos et al., 2019). For example, although they did not like the art painting, females still tended to praise the painting by telling white lies to the art student who painted it (Bell & DePaulo, 1996). Moreover, females are even willing to lie to help others at the expense of their own benefits (Erat & Gneezy, 2012). The above gender differences in the motivation and tactics of deception maybe due to the differences in preferences between genders (Croson & Gneezy, 2009). Specifically, females appear to be more altruistic or other‐regarding (Croson & Gneezy, 2009), and show higher levels of anxiety during deception than males (Vasconcellos et al., 2016; Vasconcellos et al., 2019). Compared with females, males are more likely to show competitive preference (Croson & Gneezy, 2009). As for the frequency of deceptive behavior, it varies in different deceptive circumstances. In competitive situations such as academic tests (Negre, Forgas, & Trobat, 2015; Niiya, Ballantyne, North, & Crocker, 2008; Sideridis, Tsaousis, & al Harbi, 2016), online dating (Guadagno, Okdie, & Kruse, 2012), and money allocation (Conrads et al., 2017; Dreber & Johannesson, 2008; Muehlheusser, Roider, & Wallmeier, 2015), males cheat more to surpass others and maintain their self‐esteem (Gneezy, Niederle, & Rustichini, 2003; Niiya et al., 2008). In noncompetitive situations such as conversation, females lie more to foster intimacy, especially when they know they would meet their partner again in the future (DePaulo et al., 1996; Tyler & Feldman, 2004). In summary, the prior studies reveal the gender differences in deception in many aspects. However, the previous studies mainly explore deception from the perspective of a single deceiver, limited work has been done from the perspective of dynamic interaction between deceiver and lie detector so far to explore the gender difference in deception and its possible influencing factors.

The interpersonal deception theory (IDT) highlighted that deception required interplay between humans (Burgoon & Buller, 2015; Buroon, Bulkr, Ebesu, White, & Rockwell, 1996). Both the cheaters and detectors during the deception should judge each other's mental states and actions, and make adjustments over time. Therefore, deception is a dynamic process which is enriched with interactive behaviors between cheaters and detectors. Eye contact is a powerful social cue to regulate deceptive behavior (Mann et al., 2012; Vrij, Mann, Leal, & Fisher, 2010). Looking into others' eyes could help people speculate about their intentions, feelings, and beliefs (Emery, 2000). Eye contact, on the one hand, could give the liars more cues to deceit. Many lines of evidences have shown that the liars always deliberately display more eye‐to‐eye contact with their partner when duping (Granhag & Strömwall, 2002; Jundi et al., 2013; Mann et al., 2012; Mann, Ewens, et al., 2013; Mann, Vrij, et al., 2013). On the other hand, eye contact provides the judgers with more additional cues to identify the intentions and dispositions of the liars. Frank and Ekman (2004) reported that observers' judgments depended more on facial behaviors, especially eye contact (Frank & Ekman, 2004). Maintaining eye contact could improve observers' ability to discern the truth, which makes lie‐detection easier and more accurate (Su & Levine, 2016; Vrij et al., 2010). Although many previous studies have so far revealed the key role of eye contact in deception, little is known about the details of gender difference in eye contact during deception. A few studies have shown that females might have a greater preference and advantage for eyes (Hall, Hutton, & Morgan, 2010; Proverbio, Zani, & Adorni, 2008). Females were not only significantly stronger than males in attention to gaze cue (Alwall, Johansson, & Hansen, 2010; Bayliss, Pellegrino, & Tipper, 2005), but also tended to maintain more eye contact than males in social communication with others (Exline, Gray, & Schuette, 1965). Based on these findings, we speculate that the use of eye contact in deception might also be different between males and females. Therefore, one of the goals of this study was to examine the gender difference in the role of eye contact during deception.

Numerous pieces of neuroimaging evidences have demonstrated the increased activation of the prefrontal cortex (PFC) (Fullam, McKie, & Dolan, 2009; Wright, Bishop, Jackson, & Abernethy, 2013) and right temporal parietal junction (rTPJ) (Bhatt, Lohrenz, Camerer, & Montague, 2010; Grèzes, Frith, & Passingham, 2004) during deception. However, only several studies have investigated gender difference on neural basis of deception (Gao, Yang, Shi, Lin, & Chen, 2018; Marchewka et al., 2012). For example, Marchewka et al. (2012) reported the increased left middle frontal gyrus activation in males compared to females when participants lied to answer the questions about their personal information (Marchewka et al., 2012). Enhanced right DLPFC/disrupted left DLPFC with transcranial direct current stimulation (tDCS) led to decreased deception only in females, not males (Gao et al., 2018). Those aforementioned studies only focused on brain activation of the deceivers from the perspective of single‐person neuroscience. Based on the IDT, it was impossible to fully understand the internal mechanism of interactive deception by examining only the single brain activity of participant (Hari & Kujala, 2009). Therefore, it was essential to use hyperscanning technique, which permitted us to measure two or more brains simultaneously (Montague et al., 2002), to investigate the gender difference in the brain–brain interactions underlying deception. In 2017, Zhang et al. found that higher interpersonal neural synchronization (INS) in the superior temporal sulcus was found when female dyads, but not male dyads, performed a two‐person gambling card‐game by an functional near‐infrared spectroscopy (fNIRS)‐based hyperscanning approach, which is the pioneering interbrain evidence for gender difference of deception (Zhang, Liu, Pelowski, & Yu, 2017). However, there were no information transfer and verbal communication between the two participants in such task, which provides the evidence for interbrain basis of differential interactive deception across genders with much lower ecological validity. In view of these limitations, the another goal of this study was to examine the gender difference in the deceptive behavior and the interpersonal neural basis of deception from a perspective of two‐person neuroscience by using a deceptive paradigm more closer to real life deception.

Therefore, in the present study, we attempted to elucidate gender difference of interactive deception by a face‐to‐face spontaneous sender–receiver deception task adapted from previous study (Volz, Vogeley, Tittgemeyer, von Cramon, & Sutter, 2015). In this task, two participants in each dyad sat face‐to‐face. The sender in each dyad of participants sends a message (honesty or deception) about the monetary matrix to the receiver and makes an oral statement for purpose of persuading or misleading the receiver. The receiver in each dyad will make a thorough observation or mindful listening for the sake of prevention from being deceived and make a final decision. Therefore, there are enriched interaction between two participants in each dyad during such deception task. We collected eye contacts and brain activities of the PFC and rTPJ from both participants in each dyad by combination of video recording and the fNIRS‐based hyperscanning approach.

Many neuroimaging studies using different experimental protocols exhibited a consistent conclusion that PFC played a critical role in the executive control system when deceiving (Ding, Gao, Fu, & Lee, 2013; Ding, Sai, Fu, Liu, & Lee, 2014; Greene & Paxton, 2009; Maréchal, Cohn, Ugazio, & Ruff, 2017; Tang et al., 2019) and detecting (Harada et al., 2009; Wright et al., 2013). Moreover, previous study also revealed that the PFC was associated with eye contact (Koike et al., 2016). During mutual eye contact, the brain activities of PFC between the two interacting individuals were more synchronized (Hirsch, Zhang, Noah, & Ono, 2017; Koike et al., 2016). Accordingly, it could be inferred that the real‐time deceptive interaction between the deceiver and detector may lead to brain–brain interaction in the PFC due to eye contact and the engagement of the common executive system. Based on the previous findings that females were more sensitive to eye cues (Hall et al., 2010; Proverbio et al., 2008) and had more advantages in executive control than males (Fillmore & Weafer, 2004; Rosenblitt, Soler, Johnson, & Quadagno, 2001), we speculated that the increased brain synchronization in female dyads might be more likely to occur in the PFC than male dyads during deception. As a key region of theory of mind (ToM), the rTPJ was involved in understanding and expecting the mental states and behaviors of others in deception (Leslie, 1987; Lissek et al., 2008). The increased activation in rTPJ was not only associated with the deception of the deceiver (Bhatt et al., 2010; Lisofsky, Kazzer, Heekeren, & Prehn, 2014; Tang et al., 2019), but also with the lie identification of the detector (Grèzes et al., 2004; Sowden, Wright, Banissy, Catmur, & Bird, 2015). Thus, mutual inference between the two sides may cause the brain–brain synchronization in the rTPJ. Considering the involvement of ToM in deception for both genders, we speculated that there may be the increasement of brain synchronization in rTPJ for both male dyads and female dyads during deceptive interactions.

Thus, by combining the dyadic face‐to‐face spontaneous deception paradigm and hyperscanning technique, our study aimed to: (a) identify the behavioral difference of deception between genders during a spontaneous deception, (b) explore the neural mechanisms underlying deception across different genders from a perspective of two‐person neuroscience, and (c) figure out the role of eye contact in such gender difference during interactive deception. We hypothesized that males would lie more than females based on the prior study. During deception, the increased INS in PFC which was associated with eye contact and executive function, may appear in the female dyads, while the increased INS in rTPJ may appear in both male and female dyads. This work would provide novel brain–brain evidence for the IDT and profoundly enhance our understanding of the gender difference in deception.

## 2 MATERIALS AND METHODS

### 2.1 Participants

Ninety‐eight healthy college students (46 males, 52 females, mean age: 21.3 ± 2.5 years) participated in this study. All participants were right‐handed, with normal or corrected‐to‐normal vision. They did not have any neurological, or psychiatric disorders. Participants were randomly paired into same‐gender dyads (26 female–female dyads and 23 male–male dyads), serving as sender and receiver, respectively. For each dyad, the members did not know each other before the experiment.

Written informed consent was obtained from all participants before the experiment. This study was approved by the University Committee on Human Research Protection at East China Normal University and was carried out in accordance with the approved guidelines. After the experiment, all participants could get a basic compensation of 30 RMB Yuan and additional bonus based on their performance. To be specific, one trial of payoffs was picked out randomly, and the dyad was paid in accordance with the receiver's choice in this picked trial to enhance their motivation to concentrate on the task. In total, participants were paid ranging from 30 to 60 RMB Yuan.

Data from two female dyads and two male dyads were excluded due to failure of data collection. The other two male dyads were excluded with respect to poor task comprehension. In addition, two female dyads were also excluded because of high honesty rate. Too high honesty rate would lead to too few deception trials, so that the results could not reflect the subjects' deceptive behavior well. Specifically, the honesty rate of one pair was 87.50%, and the other pair was 95.83%. Therefore, data from 22 female dyads and 19 male dyads were further analyzed in the current study.

### 2.2 Spontaneous sender–receiver deception task

**FIGURE 1:**
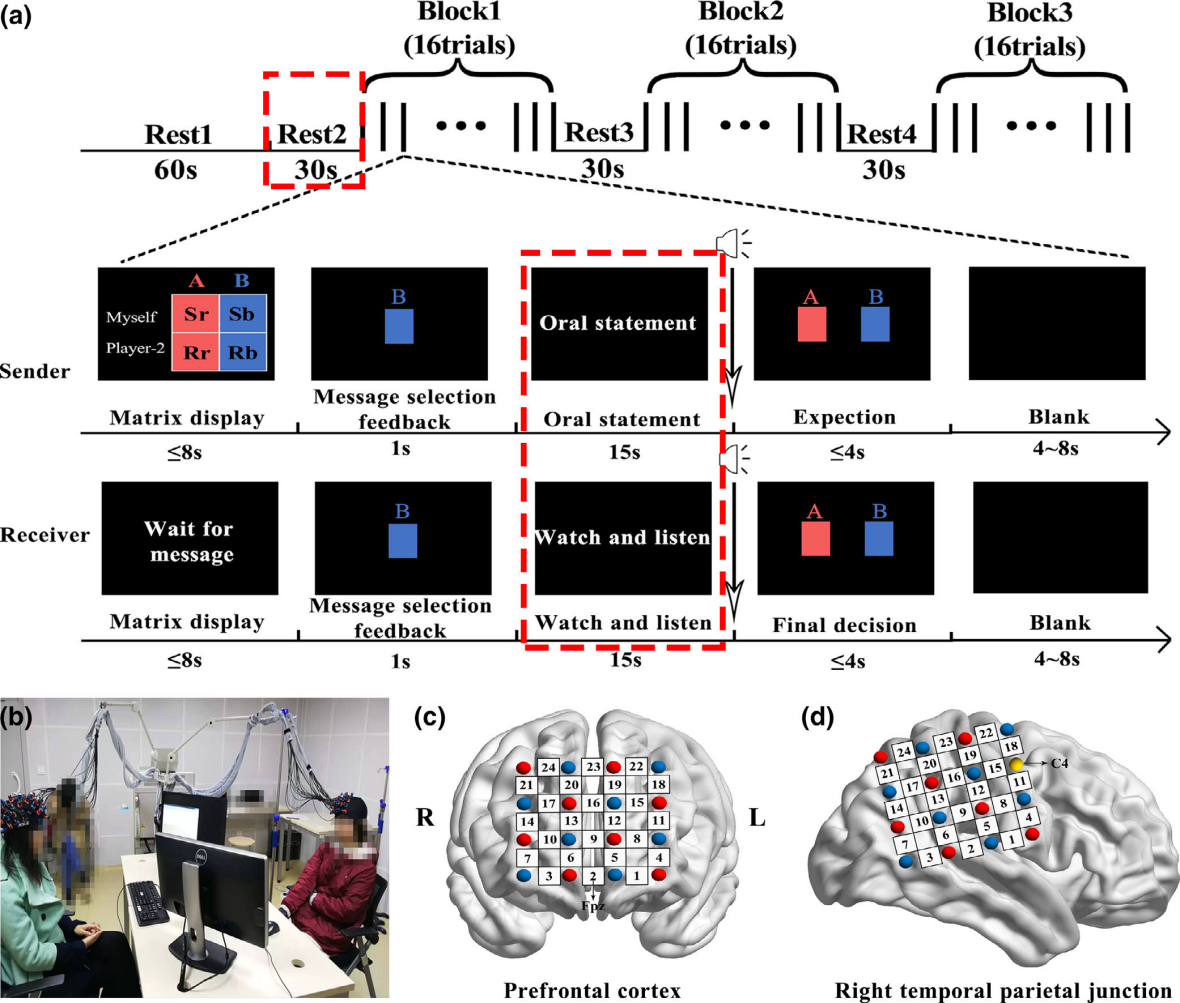
Experimental design. (a) Experimental procedures. Three blocks of spontaneous sender–receiver deception task were included in the experiment. Each block consisted of 16 trials. The analyses were focused on the Rest2 (the 30 s before the Block1, red dotted line frame) and the oral statement stage and Watch and listen stage (red dotted line frame). (b) Experimental scene. Each participant of a pair sat face to face. (c,d) Optode probe set. The optode probes were placed on the prefrontal cortex and the right temporal parietal junction. The Fpz (midpoint between the second and third probes in the lowest row of (c) and C4 (yellow circles of (d)) in the International 10–20 system were used as reference sites

The present study used a spontaneous sender–receiver deception task adapted from Volz et al. (2015). In this study, the two participants in a group sitting face‐to‐face, played different roles: sender or receiver. At the beginning of each trial, the sender could see the monetary matrix including the payoffs for himself/herself and the other under two options (A and B). As is shown in the matrix of Figure 1a, the red color represented Option A, while the blue represented Option B. The first and the second rows represented the payoffs of sender and the receiver, respectively. In other words, Option A meant that the sender got Sr (sender red) Yuan and the recipient got Rr (receiver red) Yuan. In Option B, the sender and the receiver got Sb (sender blue) Yuan and Rb (receiver blue) Yuan, respectively. It was noticeable that the payoffs between the sender and receiver for each option were conflicting. That is, if the sender got more payoffs than receiver in Option A, he/she must receive less money than receiver in Option B. The sender needed to send a message to the receiver by pressing keys (A or B): “Option A is more profitable for you” or “Option B is more profitable for you.” One of these messages was always true and the other was false based on the conflicting matrix, which was used to enhance participants' incentives to deception. Meanwhile, the receiver who could not see the matrix was informed to wait for the sender's message. If the sender chose A(B), the computer screen in front of the receiver would display the message “You will earn more money by selecting Option A(B).” Then, the sender talked to the receiver aiming to convince the receiver until the beep rang, while the latter just listened without speaking or asking to interrupt the former's monolog. The sender could say anything he/she wants on the promise that what he/she said was consistent with the message he/she sent. After the oral statement, the sender had to answer the question “which option do you expect the receiver to choose?” by pressing keys (A or B). At the same time, the receiver must make the final choice, which determined the payoffs received by both parties (Figure 1a).

In this task, the value of each option in the money matrix would affect the sender's choice. If there was a large difference between the sender's payoff and the receiver's payoff, then the sender would be more likely to deceive the receiver for his/her own benefit. We use “the tension to deceive (TD)” to define the possibility that sender would deceive the receiver under a specific matrix condition. The TD was calculated as the product of the monetary difference between the two options of the sender and the difference between the two options of the receiver, that is, (Sb–Sr) × (Rr–Rb). In the present study, the values of TD were 1, 5, 10, 25, 50, and 100 and the order of different TDs was counterbalanced across dyads to avoid the impact of TD on sender's selection (see Table S1 in supplementary materials).

In this task, the sender decided whether to lie or not spontaneously. The sender's behavior could be divided into three types: (a) honesty: the sender sent a true message and wanted the receiver to choose this option eventually; (b) deception: deception consisted of two behaviors, one was that the sender sent the false message and expected the receiver to follow it, and the other was that the sender sent the truth, but hoped the receiver chose the opposite; and (c) undefined: the sender told the false information, but expected the receiver to choose the real one. The last kind of performance (undefined) was not considered in data analysis. According to the final decision of receiver in the deception trials, the sender's lies could be divided into two types: successful deception and failed deception. If the receiver chose the option which led to more money for the sender, the sender deceived successfully in this round (successful deception); if the receiver's final choice made himself/herself benefit more in the deception trials, the sender failed to deceive (failed deception). In addition, no feedback was presented to the dyads during the experiment to eliminate the learning and order effects. The receiver could not find out whether the sender sent the truth message or not, and both the sender and receiver did not know the final payoff for each trial. That is to say, both of them did not know whether they were successful.

### 2.3 Experimental setting and procedure

Each pair of participants sat face‐to‐face. The two computer displays were placed diagonally opposite the two participants so that their views were not blocked by the computers (Figure 1b). Face‐to‐face setting which is closer to the real social interaction gave the sender and receiver more cues to deceive or detect lies. Two digital video cameras (Sony, HDR‐XR100, Sony corporation, Tokyo, Japan) were placed at opposite positions so that each of the two participants could be recorded throughout the experiment.

The task began with a 60 s resting period, in which the participants were asked to relax and keep their heads as motionless as possible (Dai et al., 2018). Thereafter, they were asked to complete the spontaneous sender–receiver deception task. As described above, the task consisted of four phases: matrix display (within 8 s), message selection feedback (1 s), oral statement (15 s), expectation and final decision (within 4 s) in order (Figure 1a). The whole experiment included three blocks and each block contained 16 trials. The intertrial interval lasted 4–8 s and the interblock interval was 30 s. In total, the whole experiment lasted around 28 min and was videotaped for subsequent coding.

### 2.4 fNIRS data acquisition

During the experiment, the imaging data from each dyad was measured simultaneously by a multichannel fNIRS system (ETG‐7100, Hitachi Medical Corporation, Japan) with a sampling rate of 10 Hz. Two 4 × 4 probe patches with a 3‐cm distance between the emitter and the detector probe were placed on the prefrontal regions for two participants in a group. The midpoint between the second and third probes in the lowest row was placed on Fpz according to the international 10–20 system. The center channel column between the second and third probe columns was placed along the sagittal reference curve (Figure 1c). The other two 4 × 4 probe patches were placed on the rTPJ for the two participants in a group, with the yellow optode placed on C4 in the International 10–20 system. The angle between the probe patches and the transverse plane was 15° (Figure 1d). In each patch, 8 emitters and 8 detectors were positioned alternatingly for a total of 16 probes, resulting in 24 measurement channels. In total, 48 channels were measured in each dyad: 24 channels in the PFC and 24 channels in the rTPJ.

The optical data in each channel was transformed into the changes in concentrations of oxy‐hemoglobin (HbO) and deoxy‐hemoglobin (HbR) based on the modified Beer–Lambert law. This study focused only on the HbO concentration, which was confirmed to be the most sensitive indicator of changes in the cerebral blood flow in fNIRS measurements (Hoshi, 2007). The correspondence between the fNIRS channels and the measurement points on the cerebral cortex was displayed in the light of the results of the virtual registration method, which had been identified by a multisubject study of anatomical craniocerebral correlation (Okamoto et al., 2004; Singh, Okamoto, Dan, Jurcak, & Dan, 2005; Tsuzuki et al., 2007).

### 2.5 Data analysis

#### 2.5.1 The performance of deception

To compare the behavioral choice between males and females, two indexes were calculated: (a) honesty rate: the percentage of the honest trials in all trials within each dyad; for subsequent analyses, dyads with high honesty rate (>85%) were removed to avoid insufficient deception trials for comparison and subsequent brain synchronization calculation. (b) Deception rate: the percentage of the deception trials in all trials within each dyad. Then, two‐way mixed repeated measures analysis of variance (ANOVA) was conducted with gender (male vs. female) as a between‐subject factor and the behavior‐type (deception rate vs. honesty rate) as a within‐subject factor. Moreover, to assess the gender differences in the ability to deceive, an additional index was calculated: (c) the success rate of deception: the percentage of the successful deception trials in all deception trials within each dyad. The success rate of deception of both genders was compared using an independent sample *t* test.

#### 2.5.2 Coding of eye contact based on video‐recording data

Four female postgraduate students who did not participate in experimental design or data collection were recruited as coders. Four female pairs were excluded to further analysis due to video‐recording failure. The eye gaze referred to the eye activities in which one of the two participants looked at the other's eye during the oral statement phase. The time points of onset and ending of each eye gaze were encoded in all dyads independently by the ELAN‐5.2 (https://tla.mpi.nl/tools/tla-tools/elan/). The eye contact in this study was defined as the intersection of two subjects' eye gaze (i.e., if Participant A had an eye gaze ranged from the 2,000 to 2,500 ms in a trial where Participant B had an eye gaze ranged from the 2,200 to 2,800 ms, then onset and ending of this eye contact was the 2,200 and 2,500 ms). The average number of eye contact in deception trials was calculated as the cumulative number of eye contact in all deception trials divided by the total number of deception trials. The average duration of eye contact in deception trials was defined as the sum of the difference between the onset and ending of each eye contact in all deception trials divided by the total number of deception trials. The intercoder reliability was 0.960 for the average number of eye contact and 0.973 for the average duration of eye contact by the intraclass correlation (Werts, Linn, & Jöreskog, 1974). The difference of the eye contact between males and females were statistically compared by the independent sample *t* test. In addition, the difference of eye contact between the successful deception and the failed deception across genders was explored by a two‐way mixed ANOVA with gender (male vs. female) as a between‐subject factor and the deception performance (successful deception vs. failed deception) as a within‐participant factor (please see SI text for details in supplementary materials).

#### 2.5.3 Relationship between deceptive behavior and eye contact

Pearson's correlation analysis was performed to analyze the relationships between the success rate of deception and eye contact indicators in deception trials (the average number and the average duration of eye contact in deception trials) separately, for different genders.

#### 2.5.4 fNIRS data analysis

During preprocessing, principle component analysis (PCA) approach was applied to remove the global (systematic) components (i.e., blood flow variation, blood pressure, respiratory) which were not task‐specific activities in fNIRS data (Zhang, Noah, & Hirsch, 2016). Then, a MATLAB package (http://grinsted.github.io/wavelet-coherence/) was used to perform wavelet transform coherence (WTC) to assess the relationship between the two HbO time series of the same channels generated by each dyad on the whole experiment (Grinsted, Moore, & Jevrejeva, 2004). Then, we identified a frequency band between 15 and 50 s (i.e., 0.02–0.07 Hz), corresponding to the duration of single trial in our task. This frequency band was more sensitive to the task (the red border line in the Figure S1) and excluded the high and low frequency physiological noises, such as cardiac pulsation (0.8–2.5 Hz) and respiration (about 0.2–0.3 Hz). Before the WTC analysis, no head motion correction was conducted in accordance with previous studies (Dai et al., 2018; Zheng et al., 2018). There were three main reasons: (a) fNIRS had a higher tolerance for motion compared to fMRI and EEG. Therefore, it was widely used to measure the brain activities of participants in action tasks, such as driving (Yoshino, Oka, Yamamoto, Takahashi, & Kato, 2013), playing the table tennis (Balardin et al., 2017), and drumming (Duan et al., 2015); (b) the frequency band we selected could avoid the high‐frequency head movements; and (c) the WTC which normalized the amplitude of signal within each time‐window was not susceptible to transient spikes caused by head motion (Nozawa, Sasaki, Sakaki, Yokoyama, & Kawashima, 2016).

Next, the INS in the oral statement phase (15 s, the red dotted line frame in the Figure 1) within deception trials and baseline (30 s resting state before the Block1, the red dotted line frame in Figure 1) were averaged separately according to the above frequency band. The deception‐related INS was defined as the INS difference of oral statement phase in deception trials relative to the baseline (i.e., deception trials–rest). Then, the INS was transformed into Fisher z‐statistics (Cui, Bryant, & Reiss, 2012). Thereafter, one‐sample *t* test for the INS was performed across each channel with false discovery rate (FDR) correction. The visualization of the above *t* test results was performed following these two steps below. The *t*‐values and MNI coordinates were first converted into an image file using xjview toolbox (nirs2img.m, http://www.alivelearn.net/xjview/), and then the transformed image file was rendered over the 3D brain model by BrainNet Viewer toolbox (http://www.nitrc.org/projects/bnv/) (Xia, Wang, & He, 2013). Moreover, an independent sample *t* test on the significant deception‐related INS between genders was also conducted. The results were corrected with the FDR method at *p* < .05 level.

#### 2.5.5 Validation by randomizing the data

To verify that INS increase came from the real interaction between each dyad in the process of deception, not by accident, a validation approach was applied. The time series of each participant in each pair was permutated 1,000 times, and then the INS data was reanalyzed. Then, two‐sample *t* test was conducted to access the difference between the reanalyzed INS and real INS at significant channel.

#### 2.5.6 Relationship between deceptive behavior and INS

To test the relationship between deceptive behavior and INS, bivariate Pearson correlation analyses between the success rate of deception and the significant deception‐related INS after the independent sample *t* test were performed in both female and male groups. In addition, the similar analyses between the significant deception‐related INS and the eye contact in deception trials were also conducted.

#### 2.5.7 Mediation effect analysis

To test whether the INS played a mediation role in the relationship between eye contact and successful deception, mediation analysis was conducted using bootstrapping method by Mplus. Bootstrapping, a nonparametric test method, which was not based on normal distribution hypothesis, had been confirmed to have lower Type I error rate and higher power in many studies (Briggs, 2006; MacKinnon, Lockwood, Hoffman, West, & Sheets, 2002; MacKinnon, Lockwood, & Williams, 2004; Williams & MacKinnon, 2008).

Bootstrapping involves resampling data repeatedly (Preacher & Hayes, 2008; Preacher, Rucker, & Hayes, 2007). First, Resampling is repeated *k* times (*k* = 10,000 in our study) from the original dataset with replacement, generating *k* new resampled samples and *k* estimates of the indirect effect. The indirect effect, denoted as *ab*, refers to the product of the coefficient *a* (the effect of the independent variable *X* on the intervening variable *M*) and the coefficient *b* (the effect of *M* on the dependent variable *Y* when *X* is statistically controlled). Next, after sorting the k estimates from smallest to largest, the 2.5 percentile and 97.5 percentile were selected to constitute a 95% confidence interval for *ab*. If the confidence interval did not include zero, then the indirect effect is statistically significant (Hayes, 2009). In our study, we used the average number (or duration) of eye contact of deception trials as independent variable *X*, the success rate of deception as dependent variable *Y*, and INS as an intervening variable *M*.

#### 2.5.8 Coupling directionality

Several methods were used in neuroimaging studies to determine the coupling direction between neural signals, such as Granger causality analysis (GCA) (Granger, 1969; Im et al., 2010; Jiang et al., 2015), phase transfer entropy (PTE) (Cao, Wang, Liu, & Alexandrakis, 2018; Hillebrand et al., 2016; Lobier, Siebenhühner, Palva, & Palva, 2014; Urquhart, Wang, Liu, Fadel, & Alexandrakis, 2020; Wang et al., 2017), permutation conditional mutual information (Abásolo, Escudero, Hornero, Gómez, & Espino, 2008; Hall & Sarkar, 2011; Li & Ouyang, 2010; Liang, Liang, Wang, Ouyang, & Li, 2015; Wen et al., 2016), and so on. In this study, the GCA method was used, because the GCA is easy to implement, and has been widely used in previous fNIRS studies to estimate the causal relationship between fNIRS time series data, such as cooperation (Cui et al., 2012), teaching (Pan, Novembre, Song, Li, & Hu, 2018), and imitation (Holper, Scholkmann, & Wolf, 2012).

GCA was first proposed by Wiener (1956) and later formalized in data analysis by Granger (1969). The principle of Granger causality can be briefly summarized as follows: for two given time series X and Y, if the variance of the prediction error for the time series Y at the current time is reduced by including historical information from the time series X in the vector autoregressive model, then the changes in X can be identified to cause the changes in Y (Granger, 1969; Im et al., 2010). In our study, the GCA was conducted for channels that showed significant INS to estimate the direction of synchronization (i.e., was the sender driving the receiver more actively or vice versa?). Here, the data we used in the GCA was the PCA‐corrected signals, because the signals were relatively stationary after the global components removing via PCA (Guo, Wu, Ding, & Feng, 2008). The main steps of the GCA is as follows: First, for the channels associated with significant increased INS, we extracted the INS of the oral statement stage within the deception trials from the whole time series. Then, all the extracted data segments of the oral stage of deception trials were concatenated. Second, a Granger Causality Estimation toolbox (https://www.dcs.warwick.ac.uk/~feng/causality.html, Guo et al., 2008; Pu et al., 2016) was used to calculate the conditional Granger Causalities in two directions: from the senders to the receivers and from the receivers to the senders. Finally, we used one‐sample *t* test to compare the differences between each direction and zero, and then used two‐sample *t* test to examine the differences between the two directions. Moreover, to further test if the results of the GCA were robust, a permutation approach was applied. The time series of each participant in each dyad was permutated 1,000 times, and then the GCA was re‐conducted based on the permutated time series.

## 3 RESULTS

### 3.1 The performance of deception

**FIGURE 2:**
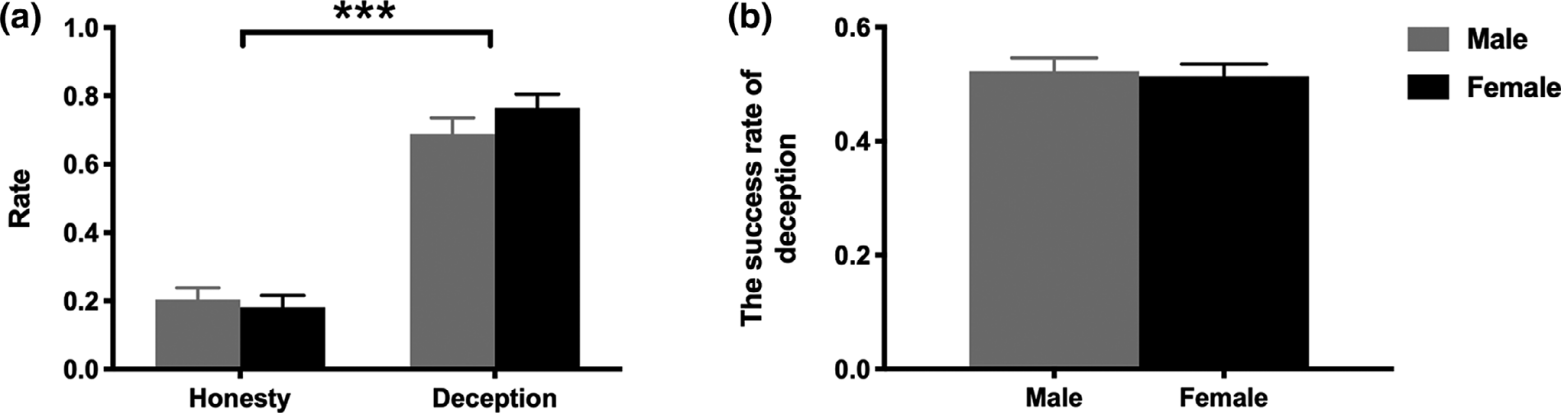
Behavioral results. (a) Behavior‐type between genders. (b) The success rate of deception between genders. Error bars indicate *SE*. ***p < .001

To examine the choice of both genders, a two‐way mixed ANOVA with the factors gender (male vs. female) and behavior‐type (honesty vs. deception) revealed a significant main effect of behavior‐type, *F*(1,39) = 99.53, *p* = .000, *η* ^2^_partial_ = 0.72, with deception rate (0.73 ± 0.20) being significantly higher than honesty rate (0.19 ± 0.16). No other significant effect (either main effect or interaction) was found (the main effect of gender: *F*(1,39) = 2.41, *p* = .13, *η* ^2^_partial_ = 0.06; the interaction effect: *F*(1,39) = 0.84, *p* = .37, *η* ^2^_partial_ = 0.02), see Figure 2a. These results indicated that deceptive behavior was induced by our experimental paradigm successfully and there was no gender difference. The following analysis focused on deception trials rather than the honest trials for two reasons: (a) the main purpose of our study was to explore the gender difference in deceptive behavior and the neural synchronization underlying deception and (b) there were very few honest trials in this study (the honesty rates were 20.43% for male pairs and 18.10% for female pairs). Independent sample *t* test on the success rate of deception showed no difference between males and females, *t*(39) = 0.28, *p* = .79, Cohen's *d* = 0.09, suggesting that the skills of deception between genders maybe comparable (Figure 2b).

### 3.2 Eye contact

The independent sample *t* test analysis revealed no difference in the averaged number or the duration of eye contact between genders (the average number of eye contact between genders: *t*(35) = −0.48, *p* = .64, Cohen's *d* = 0.16; the average duration of eye contact between genders: *t*(35) = −0.95, *p* = .35, Cohen's *d* = 0.31). These results indicated that there was no gender difference in eye contact between genders. The additional results about the difference between the successful deception and failed deception across gender could be seen in SI text and Figure S2 in supplementary materials.

### 3.3 Relationship between deceptive behavior and eye contact

**FIGURE 3:**
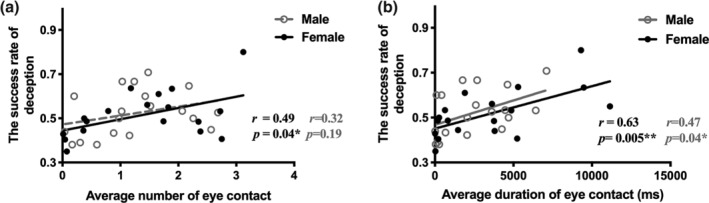
Correlations between eye contact and deceptive behavior. (a) Pearson's correlations between average number of eye contact in deception trials and the success rate of deception under different genders. (b) Pearson's correlations between average duration of eye contact in deception trials and the success rate of deception under different genders. *p < .05, **p < .01

The results of correlation analysis showed a significant positive correlation between the average number of eye contact in deception trials and the success rate of deception in female dyads, *r* = .49, *p* = .04, but not in male dyads, *r* = .32, *p* = .19, see Figure 3a. Similar analysis revealed that the average duration of eye contact was positively correlated with the success rate of deception both in females (*r* = .63, *p* = .01) and males (*r* = .47, *p* = .04), see Figure 3b.

### 3.4 INS results

**FIGURE 4:**
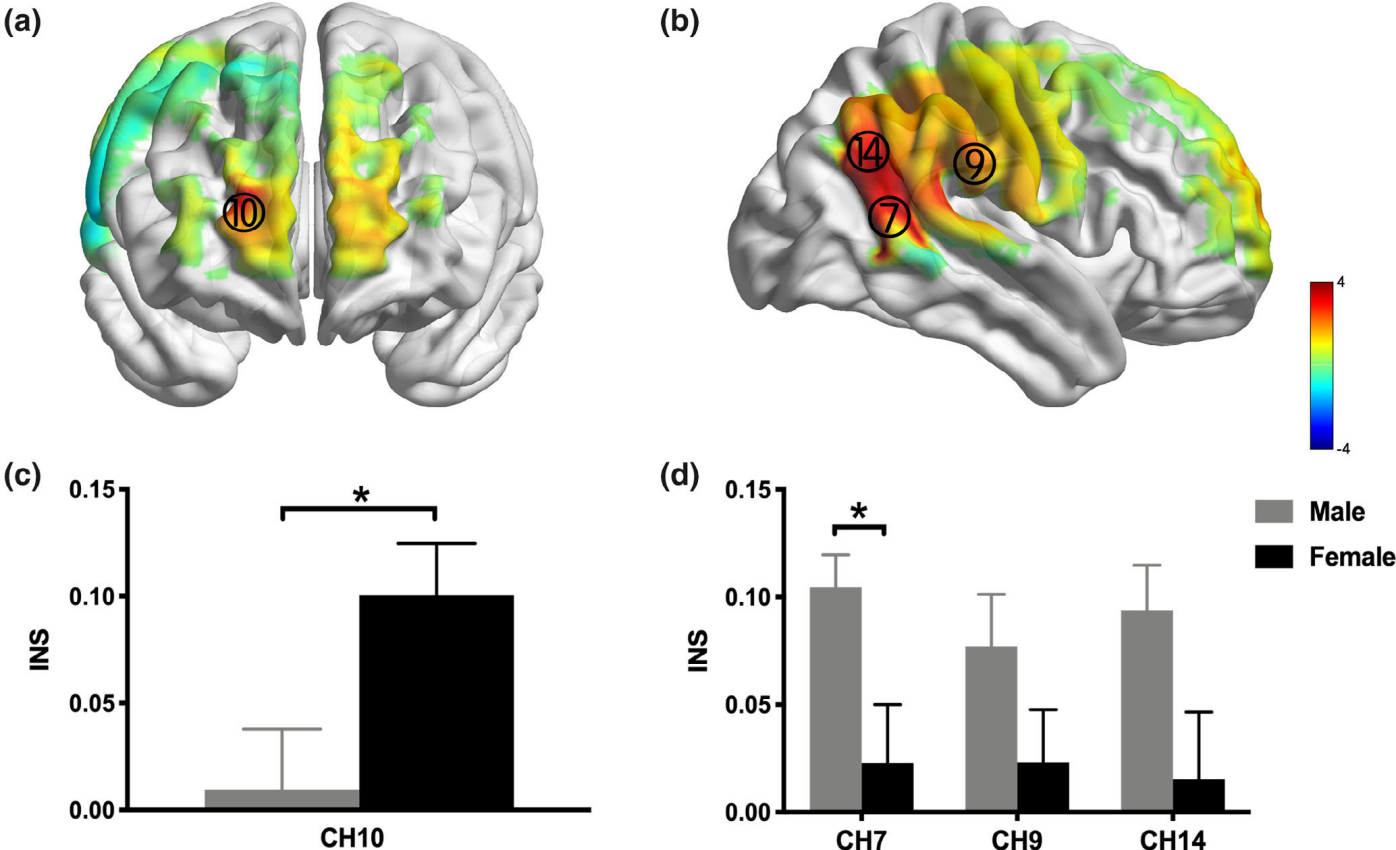
Interpersonal neural synchronization (INS) during deception trials. (a) One‐sample *t* test map of deception‐related INS for female dyads (false discovery rate [FDR] corrected). (b) One‐sample *t* test map of deception‐related INS for male dyads (FDR corrected). (c) Comparisons of INS at CH10 in prefrontal cortex (PFC) between genders. (d) Comparisons of INS at CH7, CH9, CH14 in right temporal parietal junction (rTPJ) between genders. Error bars indicate *SE*. *p < .05

One‐sample *t* test revealed that deception‐related INS was significantly increased only in CH10 in the prefrontal area for female dyads, *t*(21) = 4.15, *p* = .01, after FDR correction (Figure 4(a)). For male dyads, a significant deception‐related INS enhancement was found at three channels in the rTPJ: CH7 (*t*(18) = 6.92, *p* = .000, FDR corrected), CH9 (*t*(18) = 3.18, *p* = .04, FDR corrected), CH14 (*t*(18) = 4.49, *p* = .00, FDR corrected), see Figure 4b. The results of all channels in one‐sample *t* test of deception‐related INS can be seen in Table S2a–d in the supplementary materials. Further, the independent sample *t* tests showed that the deception‐related INS at CH10 in PFC was significantly higher in the female group than that in the male group, *t*(39) = 2.45, *p* = .02, Cohen's *d* = 0.77 (Figure 4c). Besides, the deception‐related INS for male group at CH7 in rTPJ was significantly higher than that for female group (*t*(39) = 2.51, *p* = .048, Cohen's *d* = 0.80, FDR corrected), while the difference at CH9 and CH14 did not reach significant level (CH9: *t*(39) = 1.55, *p* = .13, Cohen's *d* = 0.49, FDR corrected; CH14: *t*(39) = 2.05, *p* = .07, Cohen's *d* = 0.65, FDR corrected), see Figure 4d. In addition, for the above two significant channels (CH10 in the PFC and CH7 in the rTPJ), we further accessed the difference of INS between the successful and failed deception across gender (the details could be seen in SII text and Figure S3 in supplementary materials).

To validate the deception‐related INS, we performed the similar INS analysis on 1,000‐times permutated time series of each participant in each dyad. However, such reanalyzed INS on the randomized time series did not reach significant for male or female dyads (Figure S4a–d in supplementary materials). Two‐sample *t* tests revealed that the real deception‐related INS at CH10 was significantly higher than reanalyzed INS for female dyads (*t*(21) = 2.11, *p* = .04, Cohen's *d* = 0.68, see Figure S4e in supplementary materials) and the deception‐related INS at CH7 on the real time series was also higher than the reanalyzed INS for male dyads (*t*(18) = 3.74, *p* = .00, Cohen's *d* = 1.26, see Figure S4f in supplementary materials). The results suggested that the significantly increased INS was specific to the real deceptive interaction between dyads.

### 3.5 Relationship between INS and deceptive behavior

**FIGURE 5:**
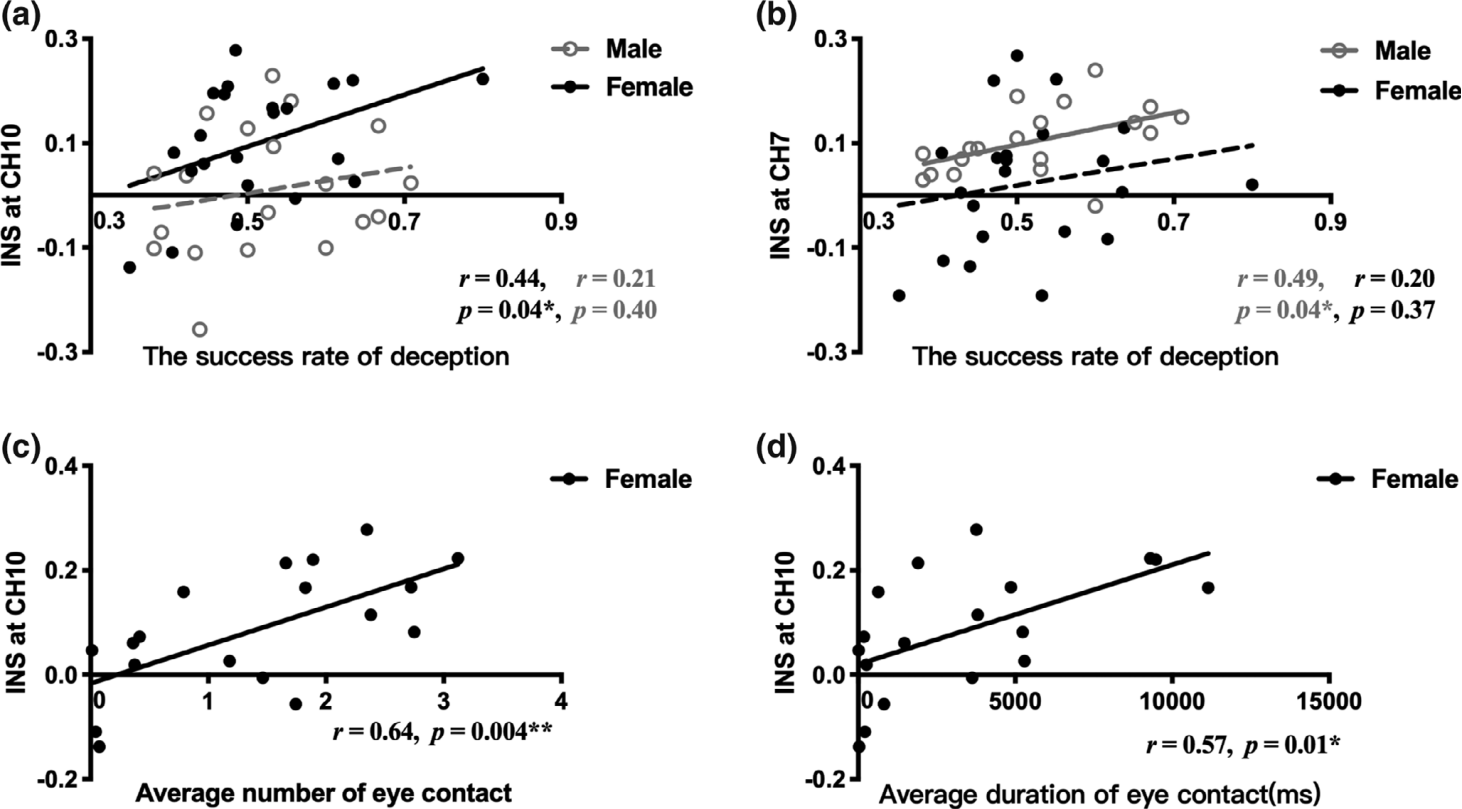
Correlations between behavioral results and interpersonal neural synchronization (INS). (a) Pearson's correlation between INS at CH10 in prefrontal cortex (PFC) and the success rate of deception for both genders. (b) Pearson's correlation between INS at CH7 in right temporal parietal junction (rTPJ) and the success rate of deception for both genders. (c) Pearson's correlation between INS at CH10 in PFC and average number of eye contact for female dyads. (d) Pearson's correlation between INS at CH10 in PFC and average duration of eye contact for female dyads. *p < .05, **p < .01

The deception‐related INS at CH10 in PFC was significantly correlated with the success rate of deception in female dyads, *r* = .44, *p* = .04, but not the male dyads, *r* = .21, *p* = .40 (Figure 5a). The deception‐related INS at CH7 in rTPJ was significantly correlated with male's success rate of deception, *r* = .49, *p* = .04, but not the female, *r* = .20, *p* = .37 (Figure 5b). These results suggested that the deception‐related INS played an important role in successful deception. The interpersonal neural basis underlying successful deception between genders were different, that is, the prefrontal synchrony might be critical to the success of deception in females, while males were tended to depend on brain synchrony in the rTPJ.

Furthermore, the deception‐related INS at CH10 in female dyads was positively correlated with both the averaged number (*r* = .64, *p* = .00, Figure 5c and duration (*r* = .57, *p* = .01, Figure 5d) of eye contact in deception trials, but there was no significant correlation in male dyads (the relationship between the INS at CH7 and the average number of eye contact: *r* = .04, *p* = .88; the relationship between the INS at CH7 and the average duration of eye contact: *r* = .17, *p* = .48).

### 3.6 Mediation effect of INS between the eye contact and the success rate of deception

**FIGURE 6:**
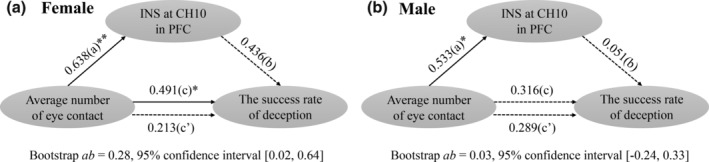
The mediation effects of interpersonal neural synchronization (INS) at CH10 in prefrontal cortex (PFC) between the average number of eye contact and the success rate of deception in both genders. (a) The mediation effect for female dyads. The effect of average number of eye contact on the success rate of deception was mediated by INS at CH10 in PFC. (b) The mediation effects for male dyads. The mediation effect was not significant. The estimates presented here were standardized coefficients. The solid and dashed lines represented significant and nonsignificant effects, respectively. (a): the effect of the average number of eye contact on the INS at CH10 in PFC; (b): the effect of the INS at CH10 in PFC on the success rate of deception when the average number of eye contact was statistically controlled; (c): the total effect of the average number of eye contact on the success rate of deception; (c′): the direct effect of the average number of eye contact on the success rate of deception when the INS at CH10 in PFC was statistically controlled. *p < .05, **p < .01

For the INS in the PFC, previous analyses in female dyads revealed the (a) positive correlation between the eye contact and the INS at CH10 in the PFC (Figure 5c,d); (b) positive correlation between the INS at CH10 in the PFC and the success rate of deception (Figure 5a); and (c) positive correlation between the eye contact and the success rate of deception (Figure 3). Based on these results, it was plausible to suppose that for females, the INS at CH10 in the PFC might play a mediation role on the effect between the eye contact and the success rate of deception. For male dyads, there were no significant increased INS at CH10 in the PFC (Figure 4b) and also no significant correlation between the INS and the success rate of deception (Figure 5a). Therefore, we speculated that there was no mediation effect of the INS in the PFC in male dyads. Indeed, the mediation analyses showed that the INS at CH10 in the PFC mediated the relationship between the average number of eye contact and the success rate of deception only in female dyads, as revealed by the bootstrap confidence interval of indirect effect which did not include zero (bootstrap *ab* = 0.28, 95% confidence interval [0.02, 0.64], Figure 6a), not in male dyads (bootstrap *ab* = 0.03, 95% confidence interval [−0.24, 0.33], Figure 6b). In addition, the mediation effects of INS at CH10 in the PFC on the relationship between the average duration of eye contact and the success rate of deception were not significant in both male and female dyads (for female dyads: bootstrap *ab* = 0.18, 95% confidence interval [−0.04, 0.49], Figure S5a in supplementary materials; for male dyads: bootstrap *ab* = −0.08, 95% confidence interval [−0.48, 0.28], Figure S5b in supplementary materials). The above results suggested that only in female dyads, the average number of eye contact predicted higher INS in the PFC, which, in turn, positively affected the success rate of deception.

For the INS in the rTPJ, although previous analyses in male dyads showed (a) positive correlation between the average duration of eye contact and the success rate of deception (Figure 3b); (b) positive correlation between the INS at CH7 in the rTPJ and the success rate of deception (Figure 5b), there was no significant correlation between the INS at CH7 in the rTPJ and the average duration of eye contact. In addition, the analyses in female dyads revealed no significant increased INS in the rTPJ. Therefore, we speculated that there was no mediation effect of INS at CH7 in the rTPJ on the relationship between the average duration of eye contact and the success rate of deception in both male and female dyads. The results of mediation analyses were consistent with our speculation (for female dyads, the indirect effect of the average duration of eye contact on the success rate of deception via the INS at CH7 in the rTPJ: bootstrap *ab* = 0.01, 95% confidence interval [−0.24, 0.21], Figure S5c in supplementary materials; for male dyads: bootstrap *ab* = 0.07, 95% confidence interval [−0.10, 0.38], Figure S5d in supplementary materials).

### 3.7 Coupling directionality

GCA was conducted on the times series of CH10 in female dyads and CH7 in male dyads to examine the direction of neural synchronization. For female dyads, the mean G‐causalities of both directions at CH10 were significantly higher than zero: from the sender to receiver (*t*(21) = 2.82, *p* = .01) and from the receiver to sender (*t*(21) = 4.57, *p* = .000). However, a two‐sample *t* test revealed that the difference of mean G‐causality between the two directions was comparable (*t*(42) = 0.16, *p* = .88, Cohen's *d* = 0.05), see Figure S6a in supplementary materials. The GCA result of male dyads was similar to female dyads. Both directions identified significant increases in the mean G‐causality relative to zero in male dyads: from the sender to receiver (*t*(18) = 2.15, *p* = .045) and from the receiver to sender (*t*(18) = 3.83, *p* = .001). Two‐sample *t* test revealed no significant difference between the two directions (*t*(36) = 1.65, *p* = .11, Cohen's *d* = 0.54), see Figure S6b in supplementary materials. These results indicated that the sender and the receiver played an equal role in the significant deception‐related INS for both genders.

To further validate the above GCA results, we reanalyzed mean G‐causalities based on the permutated time series. The two‐sample *t* tests revealed that there was no significant difference of mean G‐causality between the two directions (from the sender to the receiver; from the receiver to the sender) in both female dyads (*t*(42) = 1.26, *p* = .22, Cohen's *d* = 0.38) and male dyads (*t*(36) = 1.22, *p* = .23, Cohen's *d* = 0.40). These results further verified the equal role between the sender and the receiver for both genders.

## 4 DISCUSSION

In the current study, an fNIRS‐based hyperscanning approach was used to investigate the behavioral and interpersonal neural differences in spontaneous deception between dyadic female–female and male–male interactions. Although there were no gender difference in deception rate and the successful rate of deception, the INS underlying deception between females and males were different. That is, deception in female dyads depended on the increased INS in the PFC, while the male dyads relied on the enhancement of INS in the rTPJ. Such INS was positively correlated with the success rate of deception in both genders and had no directionality between the sender and receiver. Further, eye contact also played a different role in deception between males and females, in which only female dyads' INS in PFC mediated the relationship between the average number of eye contact and the success rate of deception. These findings provide the evidence for differential neural mechanisms underlying deception between genders from the perspective of two‐brain interaction.

### 4.1 The similar behavioral performance of deception between genders

Our study did not reveal any gender difference in deceptive behavior. We found that the success rate of deception in both genders was 51% in average and was comparable between genders. Such finding is consistent with the study of Bond Jr and DePaulo (2006), which demonstrated that humans could distinguish between truth and lies with an average accuracy of 54%, regardless of gender. In addition, the deception rate was also comparable between females and males in our spontaneous deception task, which is in line with previous studies (Childs, 2012; Gylfason, Arnardottir, & Kristinsson, 2013; Jung & Vranceanu, 2017). However, our result was in contrast to the study of Dreber and Johannesson (2008) who found that males lied more than females using the similar sender–receiver paradigm with small stake. We infer that the discrepancy between our study and Dreber and Johannesson (2008) might result from the different amount of monetary stake. That is, males lied more than females to earn a small reward, while the payoff was larger, the gender difference above disappeared. Thus, we conducted an additional ANOVA with gender as a between‐subject factor and the stake as a within‐subject factor to test this inference (see SIII text and Figure S7a,b for details in supplementary materials). The results showed no gender difference under the five stakes (the five stakes were: 1, 5, 10, 25, 50, 100), which excluded the influence of the different stake.

Thus, it appears that something else, apart from the stake may explain the inconsistency between Dreber and Johannesson (2008) and our result. The possible explanation could be the different experimental settings and individual differences. Specifically, the task in the study of Dreber and Johannesson (2008) was a one‐round spontaneous sender–receiver game, in which the deception rate was defined as the proportion of males and females who lied in the experiment. However, the task in our study contained 48 trials in each dyad and the deception rate was calculated as the percentage of the deception trials in all trials within each dyad. Besides, different personality traits, such as narcissism (Jonason, Lyons, Baughman, & Vernon, 2014), Machiavellianism (Jonason et al., 2014) and attachment styles (Ennis, Vrij, & Chance, 2008) could affect the lying frequency. The individual differences of different participants between our study and Dreber and Johannesson (2008) may be also the reason for the inconsistency results. Therefore, future studies on gender differences in deception should further explore the differences in one‐round and multirounds deceptive task, as well as the influence of personal characteristics.

### 4.2 Different interpersonal neural basis underlying deception between genders

Many previous studies on the neural basis of deception have only focused on the individual brain activity of the deceiver (Bhatt et al., 2010; Ofen, Whitfield‐Gabrieli, Chai, Schwarzlose, & Gabrieli, 2016) or detector (Grèzes et al., 2004; Wright et al., 2013), in order to explore the brain activation of the deception process or detection process. However, deception is a dynamic interactive process involving the interaction between the sender and the receiver as mentioned in IDT (Burgoon & Buller, 2015; Buroon et al., 1996). It is difficult to obtain full insight into the deceptive behavior only by measuring an isolated individual's brain activity. Therefore, we measured the brain activities of both the sender and receiver simultaneously during deception by using hyperscanning technology which has produced a wealth of findings in social interaction, such as cooperation (Cui et al., 2012), teaching (Liu et al., 2019), imitation (Holper et al., 2012), and so on.

Our study recording activities from both sender–receiver' brains have several advantages over prior single‐brain studies. The “two‐person neuroscience” in deception in our study entails the joint participation of two participants, which is closer to the deceptive behavior in real life and has higher ecological validity. Moreover, it was reported that brain‐to‐brain coupling had a higher signal‐to‐noise ratio than single‐brain recoding (Parkinson, Kleinbaum, & Wheatley, 2018). Measuring interperson brain activities which allows to assess the dynamic neural interaction between the sender and receiver, would help us to reveal the brain–brain interactive pattern of the deceptive process that could not be revealed by conventional, one person neuroimaging studies (Balconi, Pezard, Nandrino, & Vanutelli, 2017; Cui et al., 2012). Further, the sender–receiver brain coherence provides neural‐level evidence to support the IDT. That is, the INS we found during deception suggests that there is indeed interaction and interplay between the sender and the receiver in the deception process.

#### 4.2.1 The role of prefrontal INS and eye contact in deception in female dyads

For female dyads, the increased INS emerged at CH10 in PFC in our study. The CH10 was approximately located at the right superior frontal cortex (rSFC). The enhancement of INS in rSFC in female dyads played a mediation role in the effect of average number of eye contact on deception performance, which suggests that the significant INS in rSFC might result from eye contact during deception. Previous hyperscanning studies have already observed the occurrence of neural synchronization in the right frontal gyrus when following a partner's gaze toward an object (Saito et al., 2010) and gazing at each other (Koike et al., 2016). These previous studies suggest that receiving and interpreting the same eye cues may lead to the synchronization of signals across brains. In our study, eye contact, which establishes a social link between the sender and receiver (Farroni, Csibra, Simion, & Johnson, 2002), is an important nonverbal cue for dyadic deceptive interaction (DePaulo et al., 2003; Mann, Vrij, et al., 2013). During the oral statement stage in our experiment, each dyad exchanged and processed information dynamically via eye‐to‐eye contact apart from the sender's verbal content. Specifically, they could judge each other's intentions and emotional states by observing the frequency and duration of eye blink, eye avoidance, and eye contact (Marchak, 2013). This simultaneous processing of the same visual cues which arose from mutual eye contact of the sender and the receiver during deception, led to the increased INS in rSFC in our study (Hirsch et al., 2017; Koike et al., 2016). Moreover, previous studies showed that more eye contact not only gave liars more opportunities to deceive (Granhag & Strömwall, 2002; Jundi et al., 2013), but also made it easier for the detectors to lie detection (Su & Levine, 2016). Our study revealed the higher success rate of deception with more eye contact during the dynamic deceptive interaction, supporting the former view that eye contact is more useful for the deceiver in our task. The more eye contact, associated with the increased INS in rSFC, resulting in the higher success rate of deception.

#### 4.2.2 The role of INS in the rTPJ in male dyads

Different from the findings in females, the increased INS was observed at CH7 in the rTPJ in male dyads when they performed the spontaneous deception task, which indicates that such the ToM‐related brain region could play a crucial role during deception. Previous studies reveal that the TPJ including the posterior superior temporal and angular gyrus is involved in reasoning others' minds and representing the mental states of oneself and others, which is called ToM (Koster‐Hale, Saxe, Dungan, & Young, 2013; Lieberman, 2007; Saxe & Kanwisher, 2003). During the oral statement phase in deception trials in the present study, the sender inferred the receiver's belief through receiver's nonverbal cues (eye contact, facial expressions, etc.) in an effort to figure out whether the receiver believed what he/she said or not, and timely adjusted his/her actions and strategies to convince the receiver of his/her view. Similarly, the receiver might also try to deduce the sender's intention to make an informed decision by carefully listening to the sender's description, observing the sender's facial expression and making eye contact. Meanwhile, the receiver also needed to adjust his/her facial expression and eye contact to hide his/her true judgments to mislead the sender. Therefore, the INS in the rTPJ may reflect the synchronous psychological interaction caused by the mutual speculation and expectation of each other's beliefs (i.e., ToM) during deception (Zhang et al., 2017). The higher INS may indicate the stronger psychological interaction between the sender and receiver during the process of mutual speculation on each other's intentions, which was associated with higher success rate of deception. Certainly, the exact meaning of the positive correlation between the INS and success rate of deception is a direction for future research.

In summary, the present study revealed the gender difference in deception from the perspective of two‐person neuroscience. However, it was noteworthy that our findings were different from Zhang et al. (2017), which showed that the interbrain coherence in rTPJ was uniquely associated with female–female pairs but not in male–male pairs during a two‐person gambling card‐game. The inconsistency could be explained by the difference in deception paradigm and the definition of INS increase. The sender–receiver paradigm in our study included message transfer and verbal statement, which the gambling card‐game in Zhang et al. (2017) lacked. In addition, the INS in the study of Zhang et al. (2017) referred to the difference between deception‐related INS and honesty‐related INS, which highlighted the comparison between deception and honesty. The enhancement of INS was deception‐related INS in our study, which focused more on the dynamic deceptive process.

### 4.3 The equal role of the sender and receiver during deception

Our GCA results further showed that there was no significant directionality of enhanced INS in both genders, implying that the sender and receiver did not displayed any differential role during deception. These findings provide first interbrain evidence for the IDT which emphasized the important role of both the sender and receiver and their interplay during deception (Buroon et al., 1996). According to the IDT, both the sender and the receiver in deception were active participants rather than passive observers of each other's actions, and the essential attribute of deception was the interplay between the sender and receiver (Buller & Burgoon, 1996; Burgoon & Buller, 2015). In our study, the sender's verbal and nonverbal content may be influenced by receiver's facial feedback (especially eye contact). The receiver may regulate his/her facial expressions to mask or reveal their suspicions about the sender and made the final decision based on the nonverbal and verbal cues of the sender. Therefore, there is no leader–follower relationship during deception and in reality, the sender and receiver influence each other. No directionality of INS in our study highlights this point that deception is a dynamic interactive process that is not dominated by either sender or receiver and both the sender and receiver are equally important in deception (Buller, Burgoon, Buslig, & Roiger, 1996).

Our findings were inconsistent with previous studies in other social interactions. Many lines of evidence has revealed that there is a significant directional differences of INS in interactive dyads (Pan et al., 2018; Schippers, Roebroeck, Renken, Nanetti, & Keysers, 2010; Zhang et al., 2017), the primary information flows of brain coherence were from the gesturer to the guesser in a charades game (Schippers et al., 2010), from the instructor to the learner in social interactive learning task (Pan et al., 2018) and from the banker to the follower in a gambling card‐game (Zhang et al., 2017). The difference between our result and previous studies may be due to the specificity of the deception in our task. In the aforementioned studies, the participants in a pair interacted in a turn‐taking way. For example, in a charades game, the gesturer first made gestures, and then the guesser guessed (Schippers et al., 2010). Likewise, the instructor taught a song, then the learner imitated during the song learning (Pan et al., 2018). Even in a deception task, the banker checked the card and betted, then the follower decided to call or not (Zhang et al., 2017). Accordingly, the direction of the INS in these studies represented the specific information flow in the task. However, the oral statement stage in our study provides the opportunities for both the sender and receiver to interact with each other simultaneously. During this stage, the senders could use verbal and nonverbal cues to create truthful impressions. Meanwhile, the receivers could cope with the received messages and the cues to make sense the validity of those messages. Thus, nobody could dominate the face‐to‐face deception in our study, which is in line with the IDT (Burgoon & Buller, 2015).

### 4.4 Limitations

Couple pieces of limitations need to be noted in the present study. First, we found no gender difference in deception rate, which is different with some previous studies. The underlying reasons of inconsistent results need to be further explored. Second, there were not enough honest trials to be included in the data analysis due to the spontaneous deception task in our study. Further studies are needed to investigate the behavioral and neural differences between the deception and honesty. Third, although the oral statement stage was the same 15 s for each pair of participants in this study, the number and length of the sentences every sender said during this stage were different, which may have an impact on INS. Future research should attempt to control these factors. Fourth, our study revealed the association between INS and deception performance, but the causal relationship between them is still unknown, which will limit our interpretation of the brain basis underlying deception. Therefore, it is necessary for future studies to use noninvasive brain stimulation approaches, such as tDCS, tACS, and TMS to regulate INS to measure the changes of successful deception accordingly.

## 5 CONCLUSIONS

In the present study, INS could represent social interaction during the spontaneous deception. However, the enhancement of INS in the brain is significantly different between female and male dyads. Moreover, INS mediated the relationship between average number of eye contact and the success rate of deception only in female dyads. Taken together, our findings highlight the gender difference in the interpersonal neural mechanism of deception, suggesting that female deception mainly depended on eye contact compared to males, while male deception mainly entailed mentalizing relative to females. Our study advances the understanding of gender difference in sender–receiver deceptive interaction, and can be potentially used in neurofeedback during lie detection in the future.

## CONFLICT OF INTEREST

The authors declare no conflict of interest.

## Supporting information


**Appendix** **S1:** Supporting information

## Data Availability

Data in this paper will be made available on request, which permits use, adaptation and sharing. You need to indicate if changes are made and give appropriate credit to the original author(s) and the findings. If material is not included in the article, please contact the author(s) directly.
