# Supplementary material for: Neural alignment during face‐to‐face spontaneous deception: Does gender make a difference?
Source: Hum Brain Mapp. 2020 Aug 18;41(17):4964–81. doi: 10.1002/hbm.25173 (PMC7643389; doi:10.1002/hbm.25173)
Supplement: Supplementary file 1 — Appendix S1: Supporting information [file HBM-41-4964-s001.docx]

**Supplementary Material**

**Neural alignment during** **face-to-face spontaneous deception:**

**Does gender make a difference ?**

Mei Chen^1#^, Tingyu Zhang^1#^, Ruqian Zhang^1^, Ning Wang^1^, Qing Yin^1^, Yangzhuo, Li^1^, Jieqiong Liu^1^, Tao Liu^2^ and Xianchun Li^1*^

^1^ School of Psychology and Cognitive Science, Shanghai Changning-ECNU Mental Health Center, East China Normal University, Shanghai, China 200062

^2^ School of Management, Zhejiang University, Hangzhou, China 310058

# These authors contributed equally.

* Corresponding author:

Xianchun Li, School of Psychology and Cognitive Science, Shanghai Changning-ECNU Mental Health Center, East China Normal University, Shanghai, China 200062.

Email: [xcli@psy.ecnu.edu.cn](mailto:xcli@psy.ecnu.edu.cn)

**Table.S1**

Listed are all matrices in the present study (48 trials).

| Sender Red | Receiver Red | Sender Blue | Receiver Blue | TD |
| --- | --- | --- | --- | --- |
| 1 | 0 | 0 | 1 | 1 |
| 5 | 6 | 6 | 5 | 1 |
| 15 | 16 | 16 | 15 | 1 |
| 16 | 15 | 15 | 16 | 1 |
| 10 | 11 | 11 | 10 | 1 |
| 11 | 10 | 10 | 11 | 1 |
| 0 | 1 | 1 | 0 | 1 |
| 6 | 5 | 5 | 6 | 1 |
| 20 | 21 | 21 | 20 | 1 |
| 21 | 20 | 20 | 21 | 1 |
| 20 | 25 | 21 | 20 | 5 |
| 0 | 1 | 5 | 0 | 5 |
| 10 | 15 | 11 | 10 | 5 |
| 15 | 10 | 10 | 11 | 5 |
| 0 | 5 | 1 | 0 | 5 |
| 20 | 21 | 25 | 20 | 5 |
| 10 | 20 | 11 | 10 | 10 |
| 30 | 20 | 20 | 21 | 10 |
| 1 | 0 | 0 | 10 | 10 |
| 0 | 1 | 10 | 0 | 10 |
| 21 | 20 | 20 | 30 | 10 |
| 10 | 11 | 20 | 10 | 10 |
| 20 | 15 | 15 | 20 | 25 |
| 10 | 15 | 15 | 10 | 25 |
| 20 | 25 | 25 | 20 | 25 |
| 5 | 10 | 10 | 5 | 25 |
| 5 | 0 | 0 | 5 | 25 |
| 15 | 20 | 20 | 15 | 25 |
| 25 | 20 | 20 | 25 | 25 |
| 10 | 5 | 5 | 10 | 25 |
| 15 | 10 | 10 | 15 | 25 |
| 0 | 5 | 5 | 0 | 25 |
| 5 | 0 | 0 | 10 | 50 |
| 10 | 15 | 20 | 10 | 50 |
| 20 | 30 | 25 | 20 | 50 |
| 30 | 20 | 20 | 25 | 50 |
| 15 | 10 | 10 | 20 | 50 |
| 10 | 0 | 0 | 5 | 50 |
| 5 | 15 | 15 | 5 | 100 |
| 15 | 25 | 25 | 15 | 100 |
| 20 | 30 | 30 | 20 | 100 |
| 25 | 15 | 15 | 25 | 100 |
| 20 | 10 | 10 | 20 | 100 |
| 30 | 20 | 20 | 30 | 100 |
| 15 | 5 | 5 | 15 | 100 |
| 10 | 20 | 20 | 10 | 100 |
| 0 | 10 | 10 | 0 | 100 |
| 10 | 0 | 0 | 10 | 100 |

Note. TD: the tension to deceive.


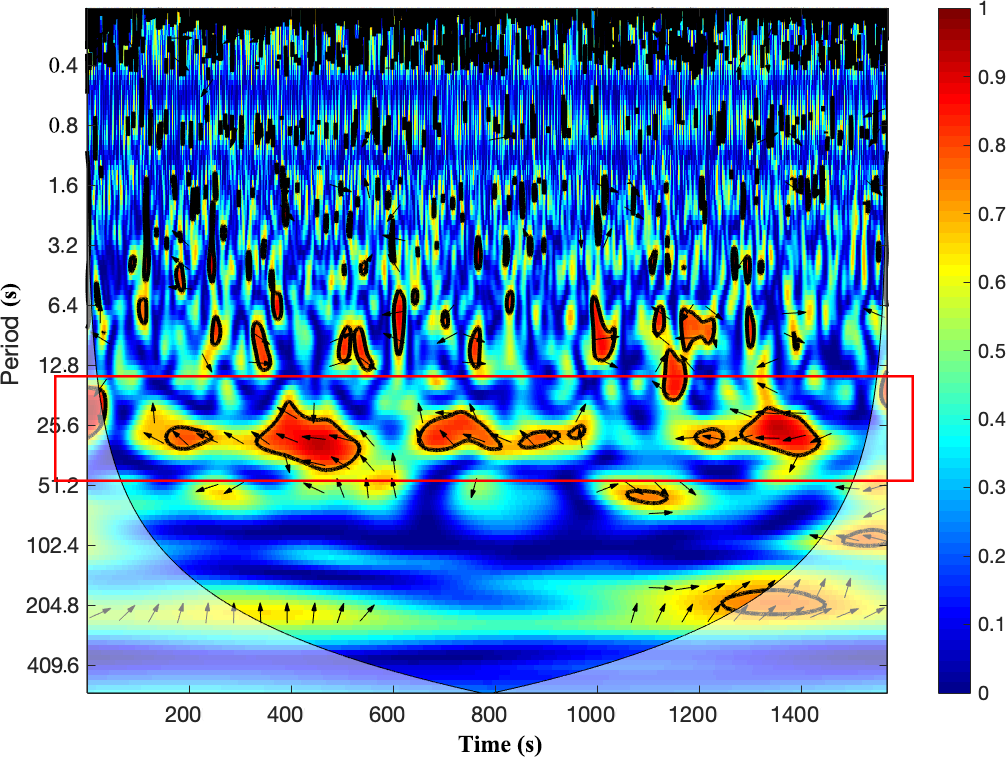


**Fig.S1.** Frequency band of interest. Wavelet transform coherence (WTC) estimating interpersonal neural synchronization (INS). The data displayed here is based on raw HbO signal from channel 9 (CH9) in prefrontal cortex (PFC) of a representative female dyad. The red border line represents the frequency band of interest (0.02–0.07Hz, 15s-50s). The cone of influence (COI) where edge effects might distort the picture is shown in faded color outside of the conical contour. The colorbar denotes the value of WTC (1 = highest coherence, 0 = lowest coherence).

**SI text**

To access the difference of eye contact between the successful deception and failed deception across gender, we calculated the cumulative number and duration of eye contact in all successful deception trials and failed deception trials separately. Then, the average number (or duration) of eye contact in successful deception trials was calculated as the cumulative number (or duration) of eye contact in all successful deception trials divided by the total number of successful deception trials. The average number (or duration) of eye contact in failed deception trials was defined as the cumulative number (or duration) of eye contact in all failed deception trials divided by the total number of failed deception trials. Finally, two 2 (gender: male vs. female) * 2 (deception performance: successful deception vs. failed deception) mixed repeated measures analyses of variance (ANOVAs) were conducted with the average number and duration of eye contact as the dependent variable separately.

For the average number of eye contact, the results showed no significant main effects (the main effect of deception performance: *F*(1,35) = 0.002, *p* = 0.96, *𝝶^2^_partial_* = 0.000; the main effect of gender: *F*(1,35) = 0.41, *p* = 0.53, *𝝶^2^_partial_* = 0.01) and interaction effect between the gender and deception performance (*F*(1,35) = 1.10, *p* = 0.30, *𝝶^2^_partial_* = 0.03), see **Fig.S2A**. For the average duration of eye contact, the results were the same. Specifically, the main effect of deception performance (*F*(1,35) = 0.63, *p* = 0.43, *𝝶^2^_partial_* = 0.02) and the main effect of gender (*F*(1,35) = 1.12, *p* = 0.30, *𝝶^2^_partial_* = 0.03) were not significant. The interaction effect between the gender and the deception performance (*F*(1,35) = 0.89, *p* = 0.35, *𝝶^2^_partial_* = 0.03) was also not significant, see **Fig.S2B**.


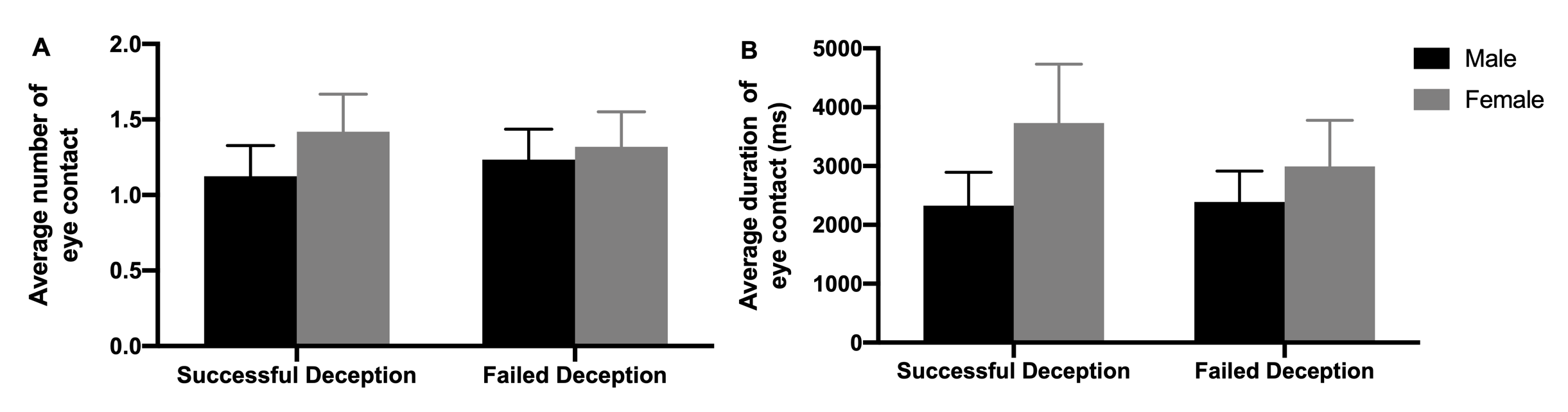


**Fig.S2.** The gender difference of eye contact between the successful and failed deception. **(A)** The gender difference of the average number of eye contact between the successful and failed deception. **(B)** The gender difference of the average duration of eye contact between the successful and failed deception. Error bars indicate standard errors.

**Table.S2A**

The results of all channels in the PFC in one sample *t*-test of deception-related interpersonal neural synchronization (INS) in female dyads.

| Channel in the PFC  in female dyads | *t*-value | *p*-value (uncorrected) | *p*-value  (FDR corrected) |
| --- | --- | --- | --- |
| 1 | 1.248 | 0.226 | 0.774 |
| 2 | 0.518 | 0.610 | 0.813 |
| 3 | 0.860 | 0.400 | 0.738 |
| 4 | 0.733 | 0.471 | 0.665 |
| 5 | 2.509 | 0.020* | 0.244 |
| 6 | 1.467 | 0.157 | 0.754 |
| 7 | 0.794 | 0.436 | 0.698 |
| 8 | 1.850 | 0.078 | 0.471 |
| 9 | 0.888 | 0.385 | 0.769 |
| 10 | 4.149 | 0.0005*** | 0.011* |
| 11 | 1.209 | 0.240 | 0.720 |
| 12 | 2.131 | 0.045* | 0.360 |
| 13 | 0.090 | 0.929 | 1.013 |
| 14 | 0.760 | 0.456 | 0.683 |
| 15 | 1.266 | 0.220 | 0.878 |
| 16 | 0.021 | 0.983 | 0.983 |
| 17 | 0.209 | 0.836 | 0.956 |
| 18 | 0.500 | 0.622 | 0.786 |
| 19 | 0.892 | 0.383 | 0.835 |
| 20 | -0.460 | 0.650 | 0.780 |
| 21 | -0.051 | 0.960 | 1.001 |
| 22 | 1.189 | 0.248 | 0.661 |
| 23 | -1.089 | 0.288 | 0.692 |
| 24 | -0.797 | 0.434 | 0.744 |

**p* < 0.05, ****p*<0.001. PFC: prefrontal cortex.

**Table.S2B**

The results of all channels in the rTPJ in one sample *t*-test of deception-related interpersonal neural synchronization (INS) in female dyads.

| Channel in the rTPJ  in female dyads | *t*-value | *p*-value (uncorrected) | *p*-value  (FDR corrected) |
| --- | --- | --- | --- |
| 1 | -1.711 | 0.102 | 0.814 |
| 2 | -0.122 | 0.904 | 0.943 |
| 3 | -0.039 | 0.969 | 0.969 |
| 4 | -0.681 | 0.503 | 0.862 |
| 5 | 1.982 | 0.061 | 0.729 |
| 6 | -0.611 | 0.548 | 0.877 |
| 7 | 0.837 | 0.412 | 0.899 |
| 8 | -0.812 | 0.426 | 0.852 |
| 9 | 0.939 | 0.358 | 1.075 |
| 10 | 0.452 | 0.656 | 0.787 |
| 11 | 0.553 | 0.586 | 0.827 |
| 12 | 0.468 | 0.645 | 0.814 |
| 13 | 1.026 | 0.317 | 1.085 |
| 14 | 0.491 | 0.629 | 0.838 |
| 15 | 2.433 | 0.024* | 0.576 |
| 16 | 1.587 | 0.127 | 0.765 |
| 17 | 0.689 | 0.499 | 0.921 |
| 18 | -0.195 | 0.848 | 0.969 |
| 19 | -0.136 | 0.893 | 0.975 |
| 20 | 0.925 | 0.365 | 0.974 |
| 21 | -0.590 | 0.562 | 0.843 |
| 22 | 1.321 | 0.201 | 0.964 |
| 23 | 0.839 | 0.411 | 0.986 |
| 24 | 1.279 | 0.215 | 0.859 |

**p* < 0.05. rTPJ: right temporal-parietal region.

**Table.S2C**

The results of all channels in the PFC in one sample *t*-test of deception-related interpersonal neural synchronization (INS) in male dyads.

| Channel in the PFC  in male dyads | *t*-value | *p*-value (uncorrected) | *p*-value  (FDR corrected) |
| --- | --- | --- | --- |
| 1 | 0.672 | 0.510 | 0.816 |
| 2 | 1.165 | 0.259 | 1.038 |
| 3 | 0.694 | 0.496 | 0.851 |
| 4 | 0.489 | 0.631 | 0.946 |
| 5 | -0.837 | 0.414 | 0.902 |
| 6 | -0.167 | 0.869 | 1.043 |
| 7 | -0.035 | 0.973 | 0.973 |
| 8 | 0.811 | 0.428 | 0.856 |
| 9 | 3.413 | 0.003** | 0.075 |
| 10 | 0.334 | 0.742 | 1.047 |
| 11 | -1.192 | 0.249 | 1.193 |
| 12 | 1.719 | 0.103 | 1.232 |
| 13 | 1.146 | 0.267 | 0.915 |
| 14 | 1.349 | 0.194 | 1.164 |
| 15 | 0.079 | 0.938 | 0.979 |
| 16 | 0.777 | 0.447 | 0.826 |
| 17 | 0.081 | 0.936 | 1.021 |
| 18 | -0.325 | 0.749 | 0.998 |
| 19 | 1.110 | 0.282 | 0.845 |
| 20 | 1.377 | 0.185 | 1.483 |
| 21 | -0.199 | 0.844 | 1.066 |
| 22 | 0.925 | 0.367 | 0.882 |
| 23 | 1.081 | 0.294 | 0.784 |
| 24 | -0.138 | 0.892 | 1.019 |

***p*<0.01. PFC: prefrontal cortex.

**Table.S2D**

The results of all channels in the rTPJ in one sample *t*-test of deception-related interpersonal neural synchronization (INS) in male dyads.

| Channel in the rTPJ  in male dyads | *t*-value | *p*-value (uncorrected) | *p*-value  (FDR corrected) |
| --- | --- | --- | --- |
| 1 | 1.795 | 0.090 | 0.239 |
| 2 | -1.144 | 0.268 | 0.357 |
| 3 | -1.087 | 0.291 | 0.368 |
| 4 | 1.211 | 0.242 | 0.341 |
| 5 | 2.654 | 0.016* | 0.077 |
| 6 | -0.790 | 0.440 | 0.503 |
| 7 | 6.923 | 0.0000*** | 0.0000*** |
| 8 | 1.611 | 0.125 | 0.249 |
| 9 | 3.175 | 0.005** | 0.042* |
| 10 | 2.818 | 0.011* | 0.068 |
| 11 | 1.628 | 0.121 | 0.264 |
| 12 | 1.946 | 0.067 | 0.202 |
| 13 | 1.248 | 0.228 | 0.342 |
| 14 | 4.490 | 0.0003*** | 0.003** |
| 15 | -0.481 | 0.636 | 0.636 |
| 16 | 1.464 | 0.160 | 0.275 |
| 17 | 2.268 | 0.036* | 0.123 |
| 18 | 0.869 | 0.397 | 0.476 |
| 19 | 1.300 | 0.210 | 0.336 |
| 20 | 1.718 | 0.103 | 0.247 |
| 21 | 0.672 | 0.510 | 0.556 |
| 22 | 1.523 | 0.145 | 0.268 |
| 23 | 0.507 | 0.618 | 0.645 |
| 24 | 2.320 | 0.032* | 0.129 |

**p* < 0.05, ***p*<0.01, ****p*<0.001. rTPJ: right temporal-parietal region.

**SII text**

To access the difference of significant INS between the successful and failed deception across gender, we calculated the successful deception-related INS and the failed deception-related INS for the significant channels (CH10 in the PFC and CH7 in the rTPJ) separately. The successful deception-related INS was defined as the INS difference of oral statement stage in successful deception trials relative to the baseline (i.e., successful deception trials–rest). Similarly, the failed deception-INS was defined as the INS difference of oral statement phase in failed deception trials relative to the baseline (i.e., failed deception trials–rest). Then, a 2 (gender: male vs. female) * 2 (deception performance: successful deception vs. failed deception) ANOVA was conducted with INS in CH10 and INS in CH7 as the dependent variable separately.

For the CH10 in the PFC, the results showed the main effect of gender (*F*(1,39) = 5.66, *p* = 0.02, *𝝶^2^_partial_* = 0.13), with the INS of female dyads (0.10 ± 0.11) being significantly higher than that of male dyads (0.01 ± 0.12). No other significant effect (either main effect of deception performance or interaction) was found (the main effect of deception performance: *F*(1,39) = 0.01, *p* = 0.92, *𝝶^2^_partial_* = 0.00; the interaction effect: *F*(1,39) = 0.00, *p* = 0.96, *𝝶^2^_partial_* = 0.00), see **Fig.S3A**. For the CH7 in the rTPJ, the main effect of gender was also found (*F*(1,39) = 6.24, *p* = 0.02, *𝝶^2^_partial_* = 0.14). The INS of male dyads (0.10 ± 0.07) was significantly higher than that of female dyads (0.02 ± 0.13). The main effect of deception performance (*F*(1,39) = 2.88, *p* = 0.10, *𝝶^2^_partial_* = 0.07) and the interaction between the gender and the deception performance (*F*(1,39) = 0.04, *p* = 0.84, *𝝶^2^_partial_* = 0.00) were not significant, see **Fig.S3B**.


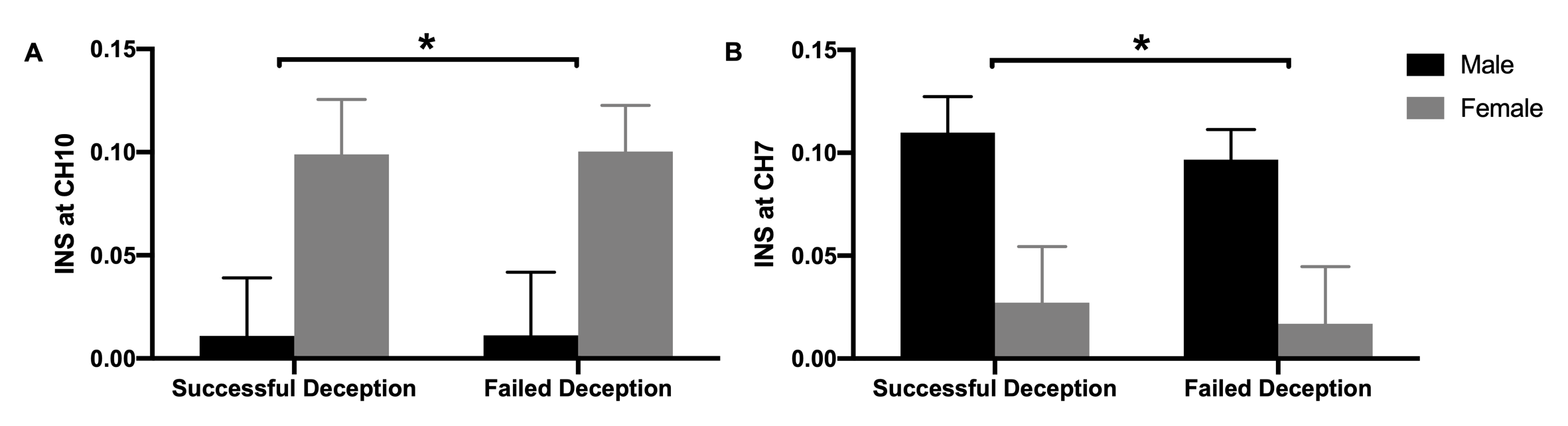


**Fig.S3.** The gender difference of significant INS between the successful and failed deception. **(A)** The gender difference of INS at CH10 in the PFC between the successful and failed deception. **(B)** The gender difference of INS at CH7 in the rTPJ between the successful and failed deception. Error bars indicate standard errors. **p* < 0.05.


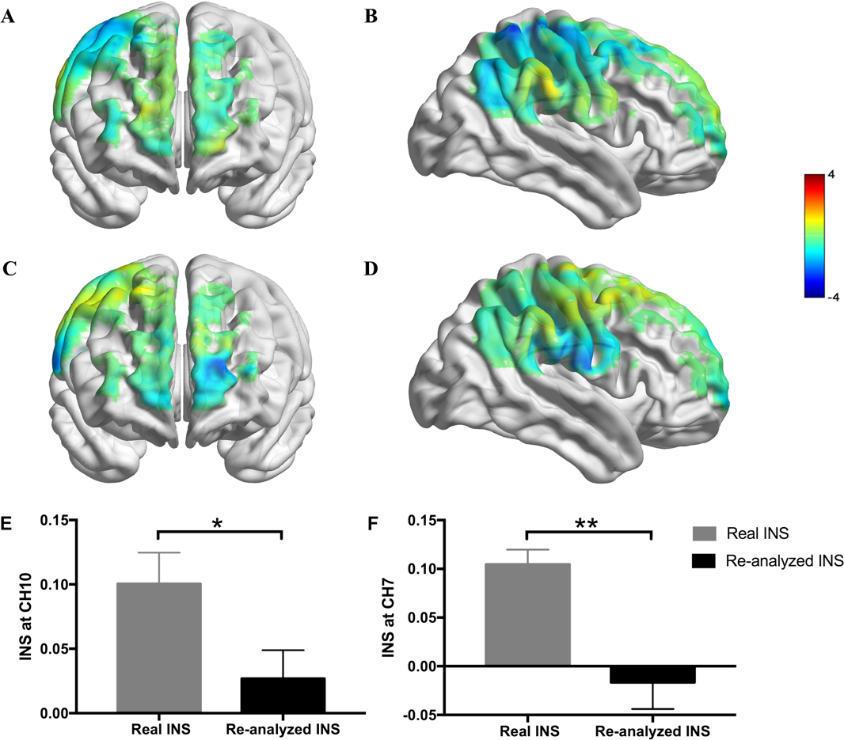


**Fig.S4.** Permutation test. **(A)** and **(B)** One sample *t*-test map of interpersonal neural synchronization (INS) for the permutated time series of female dyads in the PFC and the rTPJ separately during deception. **(C)** and **(D)** One sample *t*-test map of INS for the permutated time series of male dyads in the PFC and the rTPJ separately during deception. **(E)** Comparison between real INS and re-analyzed INS at CH10 in PFC for female dyads. **(F)** Comparison between real INS and re-analyzed INS at CH7 in rTPJ for male dyads. **p* < 0.05, ***p*<0.01.


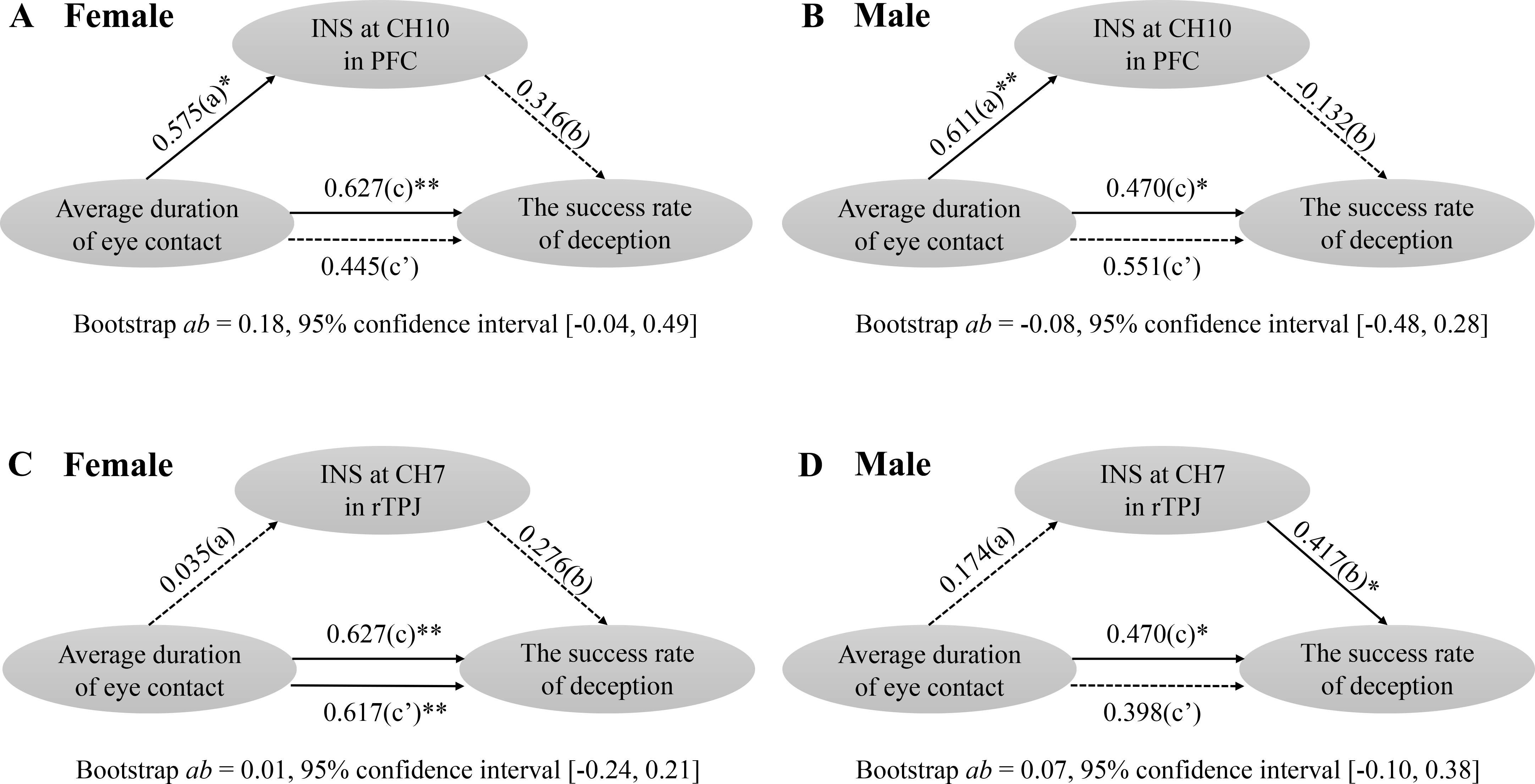


**Fig.S5.** The mediation effects. **(A)** and **(B)** The mediation effect of INS at CH10 in PFC between the average duration of eye contact and the success rate of deception for female dyads **(A)** and male dyads **(B)**; **(C)** and **(D)** The mediation effect of INS at CH7 in rTPJ between the average duration of eye contact and the success rate of deception for female dyads **(C)** and male dyads **(D)**. a: the effect of the average duration of eye contact on the INS; b: the effect of the INS on the success rate of deception when the average duration of eye contact was statistically controlled; c: the total effect of the average duration of eye contact on the success rate of deception; c’: the direct effect of the average duration of eye contact on the success rate of deception when the INS was statistically controlled. The estimates presented here were standardized coefficients. The solid and dashed lines represented signiﬁcant and non-signiﬁcant effects, respectively. **p* < 0.05, ***p* < 0.01.

**
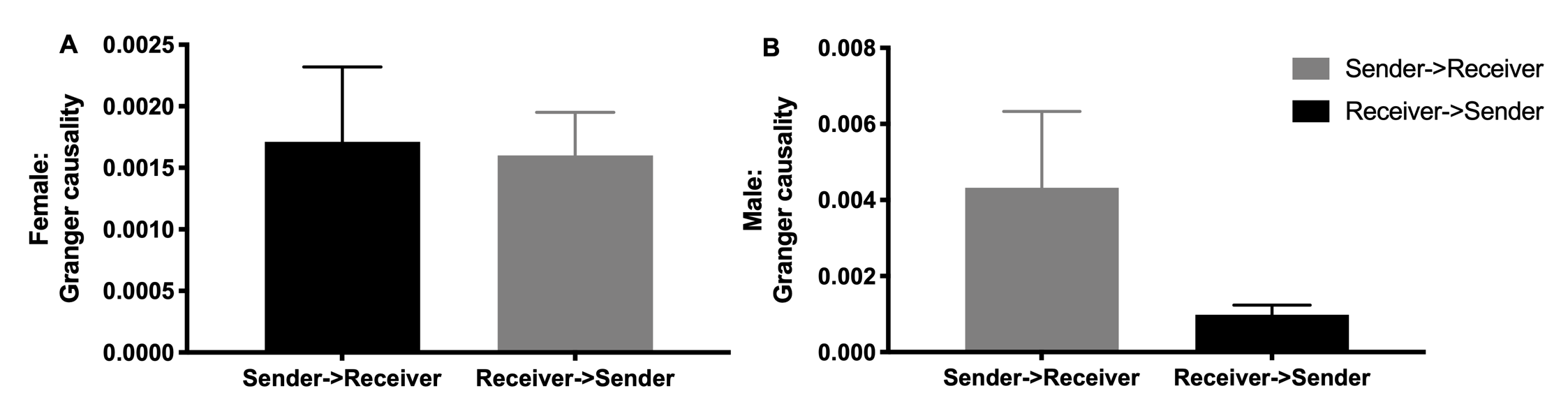
**

**Fig.S6.** Granger causality analysis (GCA) results for both genders during deception. **(A)** Comparison of mean G-causality between the two directions (from sender to receiver; from receiver to sender) for female dyads based on the real time series. **(B)** Comparison of mean G-causality between the two directions (from sender to receiver; from receiver to sender) for male dyads based on the real time series.

**SIII text**

In the present study, we used “the tension to deceive (TD)” to represent the stake, because TD could take into account the monetary benefits of both the sender and the receiver. The values of TD were 1, 5, 10, 25, 50, 100 in our study. To access the gender difference in deception rate across stakes, the two-way mixed repeated measures analysis of variance (ANOVA) was conducted with gender (male vs. female) as a between-subject factor and the stake (TD: 1 vs. 5 vs. 10 vs. 25 vs. 50 vs. 100) as a within-subject factor. The dependent variable was the deception rate, which was calculated as the proportion of deception trials under each TD condition (TD = 1 or 5 or 10 or 25 or 50 or 100) to the total trials under each TD condition within each dyad. The results showed a significant main effect of the stake, *F*(5,195) = 9.30, *p* = 0.000, *𝝶^2^_partial_* = 0.19. The higher the stake, the higher deception rate. No other significant effect (either the main effect or interaction effect) was found (the main effect of gender: *F*(1,39) = 1.63, *p* = 0.21, *𝝶^2^_partial_* = 0.04; the interaction effect: *F*(5,195) = 1.44, *p* = 0.21, *𝝶^2^_partial_* = 0.04), see **Fig.S7A**.

We also conducted the similar ANOVA with the success rate of deception as the dependent variable. The success rate of deception was calculated as the percentage of the successful deception trials under each TD condition in total deception trials under each TD condition within each dyad. No significant effects were found (the main effect of the stake: *F*(5,195) = 1.35, *p* = 0.24, *𝝶^2^_partial_* = 0.03; the main effect of the gender: *F*(1,39) = 0.03, *p* = 0.86, *𝝶^2^_partial_* = 0.00; the interaction effect between the stake and gender: *F*(5,195) = 0.70, *p* = 0.63, *𝝶^2^_partial_* = 0.02, **Fig.S7B**).


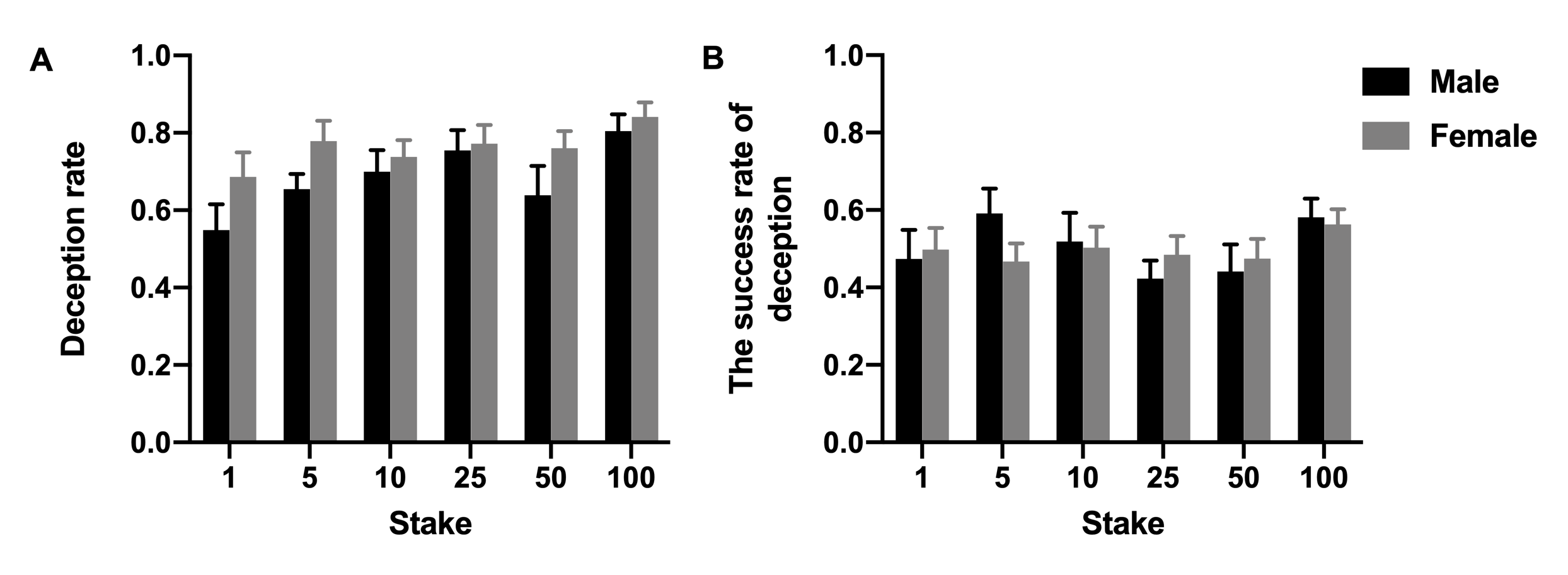


**Fig.S7.** The difference of deception across stakes. **(A)** The difference of the deception rate across stakes between genders. **(B)** The difference of the success rate of deception across stakes between genders. Error bars indicate standard errors.
